# Enhanced antibody-antigen structure prediction from molecular docking using AlphaFold2

**DOI:** 10.1038/s41598-023-42090-5

**Published:** 2023-09-13

**Authors:** Francis Gaudreault, Christopher R. Corbeil, Traian Sulea

**Affiliations:** 1 Human Health Therapeutics Research Centre, National Research Council Canada, 6100 Royalmount Avenue, Montreal, QC H4P 2R2 Canada; 2 Institute of Parasitology, McGill University, 21111 Lakeshore Road, Sainte-Anne-de-Bellevue, QC H9X 3V9 Canada

**Keywords:** Protein structure predictions, Machine learning

## Abstract

Predicting the structure of antibody-antigen complexes has tremendous value in biomedical research but unfortunately suffers from a poor performance in real-life applications. AlphaFold2 (AF2) has provided renewed hope for improvements in the field of protein–protein docking but has shown limited success against antibody-antigen complexes due to the lack of co-evolutionary constraints. In this study, we used physics-based protein docking methods for building decoy sets consisting of low-energy docking solutions that were either geometrically close to the native structure (positives) or not (negatives). The docking models were then fed into AF2 to assess their confidence with a novel composite score based on normalized pLDDT and pTMscore metrics after AF2 structural refinement. We show benefits of the AF2 composite score for rescoring docking poses both in terms of (1) classification of positives/negatives and of (2) success rates with particular emphasis on early enrichment. Docking models of at least medium quality present in the decoy set, but not necessarily highly ranked by docking methods, benefitted most from AF2 rescoring by experiencing large advances towards the top of the reranked list of models. These improvements, obtained without any calibration or novel methodologies, led to a notable level of performance in antibody-antigen unbound docking that was never achieved previously.

## Introduction

Three-dimensional structures of antibody-antigen complexes can be predicted computationally using physics-based protein–protein docking methods^1–5^. Recent benchmark studies have shown that reconstituting the complex by docking the antibody and antigen structures separated from the complex, while keeping their protein backbone conformations as in the bound state, is now a relatively trivial task and mostly a solved problem^4,6^. However, antibody-antigen docking remains significantly challenging in the more realistic scenario in which the backbone conformations of the antibody and antigen structures deviate from their bound-state conformations. In this so-called “unbound docking”, the best-ranked model achieves no more than 20% success in predicting a complex structure reasonably close to the native structure^4,6^. Encouragingly, these docking methods are relatively good at sampling various binding modes and able to enrich with native-like docking solutions a relatively small fraction of the best-scored 100–1000 poses out of billions of theoretical ones. The general failure of protein–protein unbound docking thus appears to be caused not only by the high dimensionality of the search space associated with backbone sampling^7^, but also to an inability to accurately score and rank docked structures that deviate substantially from the native geometry of the complex. The problem is further exacerbated in modeling antibody-antigen complexes due to the antibody CDR-H3 hypervariable loop which is capable of exploring various backbone conformations in the unbound versus bound states^8,9^. In our view, a step forward in the field of protein–protein and antibody-antigen docking would be to employ a complementary method to improve the scoring component. Hence, an objective of this study was to explore a way to “rescue” native-like docking structures and top-rank them ideally among the 1–5 best scoring solutions.

With the release of AlphaFold2 (AF2), artificial intelligence and deep-learning have made a breakthrough towards addressing the protein folding problem^10^. Undoubtedly, AF2 has delivered an astonishing performance in the recent blind competition CASP14 by outperforming the previously considered state-of-the-art physics-based protein structure prediction methods^11^. One interesting feature of AF2 structure predictions is that they come with confidence levels for different regions of the modeled protein structure. Most of the success and boost in performance of AF2 over its predecessor AlphaFold^12^ can be attributed to the inclusion of a Multiple-Sequence-Alignment (MSA) module in addition to the Structure module. The sequence co-evolutionary information that comes with the MSA module embeds structural patterns that strongly influence the thermostability of proteins in general^13^ and also of antibodies^14^. Thus, providing co-evolutionary information has become a key element towards accurately predicting how proteins fold or arrange themselves within multi-protein complexes^15–17^.

Many groups have used AF2 to predict the structure of complexes with the aid of co-evolutionary data. One simple approach consists in tweaking AF2 by adding a long linker between the interacting proteins. Independent releases of AF2 were developed for that specific purpose and were shown to be promising^18,19^. For instance, the performance of AF2-Multimer was shown to significantly outperform the traditional physics-based approaches in protein–protein docking^19^ when looking only at the top-scoring model. With the aid of AF2, unbound docking has made a huge step forward for those complexes that co-evolved together. However, the requirement for co-evolution data for multimeric units limits its use for predicting antibody-antigen complexes. The strong binding of antibodies towards their antigen is not a result of co-evolution but a result of somatic hypermutation and affinity maturation^20^. On this note, a study has shown that the performance of AF2-Multimer against antibody-antigen complexes is no better than the one of physics-based methods^21^, indicating the relevancy and need of physics-based methods. While other deep-learning technologies that were developed circumvent the need of the MSA component^22,23^, their performance in antibody-antigen structure prediction still remains unproven.

Several groups have explored the idea of providing structural templates obtained from various sources to AF2 for improved modeling accuracies^10^. For instance, Terwilliger et al*.* showed that they could turn initial poor-resolution experimentally-guided models into highly accurate ones by iteratively running AF2 and re-inserting the predicted models as templates in the following iterations^24^. Several other examples have been reported where AF2 was coupled instead with structure prediction tools when experimental data was not available. In those instances, structural hypotheses of partner proteins alone or in complex were generated using physics-based tools and then parsed through AF2 to assess their plausibility. For instance, Ghani et al*.* demonstrated that by starting from AF2 protein–protein complex models they could improve accuracies by redocking with ClusPro with a further refinement and scoring using AF2^25^. The authors not only noticed improved success and enrichment in the top-ranked fractions but also noticed an improved quality of their models through a structural refinement by AF2. However, their dataset contained a limited number of antibody structures which AF2 is known to have difficulty in predicting correctly on their own or in complex making prediction with ClusPro even more difficult. Another group has used AF2 to assess the plausibility and quality of protein folding models generated by Rosetta^26^. An important aspect of their study was bypassing the MSA module. By doing so, no co-evolutionary data was used in the confidence scoring, hence the resulting confidence metrics were based solely on the structure template from the Structure module alone. The authors showed significantly better discrimination of native versus non-native protein structures when folding models were re-ranked using structure-only AF2 confidence metrics relative to the Rosetta physics-based scoring function. These remarkable results obtained with an AF2 version lacking co-evolutionary information opened new possibilities for improving scoring protein structure models generated by physics-based methods.

Our present study, which focuses on the structure prediction of antibody-antigen binding for which no co-evolutionary data exists, is thus inspired and builds upon the aforementioned protein folding study of Roney et al.^26^. Here, we assessed the plausibility of antibody-antigen interaction models generated using protein–protein docking methods. We investigated if there are benefits in using AF2 in discriminating near-native structures from challenging decoy structures. To ensure consistency, the decoys were generated using three docking methods that are complementary in nature: ProPOSE, a direct-search approach that employs stiff potentials^4^, and ZDOCK and PIPER, two Fast Fourier-Transform (FFT)-based approaches employing soft potentials^2,3^. To compensate for the rigid-body nature of docking algorithms, the docking-generated models often go through a second stage of processing and reranking to improve the enrichment of near-native structures^27–29^. Hence, ClusPro was used in this study to cluster models produced by PIPER to identify popular binding modes in order to examine if antibody-antigen docking models that went through further processing also benefitted from AF2 final treatment. Two sets of decoys were generated containing structures with bound and unbound backbone conformations. The “bound-backbone” set of structures, used as control, allowed us to assess the performance of AF2 in a scenario generally more trivial for docking and scoring. The “unbound-backbone” set of structures, on the other hand, is more relevant for real-life applications but considerably more challenging. Lastly, to ensure that our findings were not biased by the fact that the interacting proteins in these sets may have been part of the very large data sets employed for training the AF2 model, a separate “bound-backbone” set comprised of more recent antibody-antigen complex structures made available after the development of AF2 was also tested here for providing an additional layer of validation.

## Results

### Decoys sets

Antibody-antigen docking simulations were performed with the ProPOSE^4^, ZDOCK^2^ and PIPER^3^ methods using as input the protein structures in their bound-backbone and unbound-backbone conformations (see Methods). The top 100 models predicted by each method were collected. On average, 33 models per system were predicted following post-processing of PIPER models with ClusPro. In total, 76,383 and 8246 models were generated from 231 and 25 unique systems for the bound-backbone and unbound-backbone decoy sets, respectively (Table 1).

**Table 1:** Composition of the bound-backbone set and unbound-backbone set of antibody-antigen complexes used in this study.

Method	Systems^a^	Models^b^	Positives (%)^c^	Negatives	P/S^d^
Bound-backbone					
ProPOSE	231	22,515	868 (3.9%) **974 (4.3%)**	21,647 **21,541**	3.8 ± 3.5 **4.2 ± 4.3**
ZDOCK	231	23,056	629 (2.7%) **740 (3.2%)**	22,427 **22,316**	2.7 ± 5.5 **3.2 ± 5.9**
PIPER	231	23,100	2266 (9.8%) **2996 (13.0%)**	20,834 **20,104**	9.8 ± 20.0 **13.0 ± 21.9**
ClusPro	231	7712	168 (2.2%) **264 (3.4%)**	7544 **7448**	0.7 ± 1.0 **1.1 ± 1.1**
Unbound-backbone					
ProPOSE	25	2500	30 (1.2%) **34 (1.4%)**	2470 **2466**	1.2 ± 1.8 **1.4 ± 2.2**
ZDOCK	25	2500	62 (2.5%) **63 (2.5%)**	2438 **2437**	2.5 ± 3.8 **2.5 ± 3.9**
PIPER	25	2440	179 (7.3%) **236 (9.7%)**	2261 **2204**	7.2 ± 15.9 **9.4 ± 17.0**
ClusPro	25	806	20 (2.5%) **29 (3.6%)**	786 **777**	0.8 ± 1.5 **1.2 ± 1.6**

^a^Systems for which the docking method produced results without technical error.

^b^Docking methods do not necessarily output 100 models even when requested by the user.

^c^Based on the docking-generated models (normal typeface) or AlphaFold2-reconstructed models (bold typeface), and models of at least acceptable quality according to CAPRI classification^30^.

^d^Mean and standard deviation for the number of positives per system (P/S).

Models were compared to their respective crystal structures and attributed a quality as previously described in the CAPRI blind challenges^30^. The models with high, medium or acceptable quality were reported in the “positives” set as opposed to the incorrectly predicted models reported in the “negatives" set. The various docking methods produced variable fractions of positives (1–13%) for a total of 3,931 and 291 positives, which came along with 72,452 and 7,955 negatives, for the bound-backbone and unbound-backbone sets, respectively. On average, 1–10 positives per system were generated. The uncharacteristically high numbers for positives per system generated by PIPER are due to redundancy within the top-100 models, which are typically subsequently clustered with the ClusPro method.

### Quality of AlphaFold2-generated models

**Figure 1:**
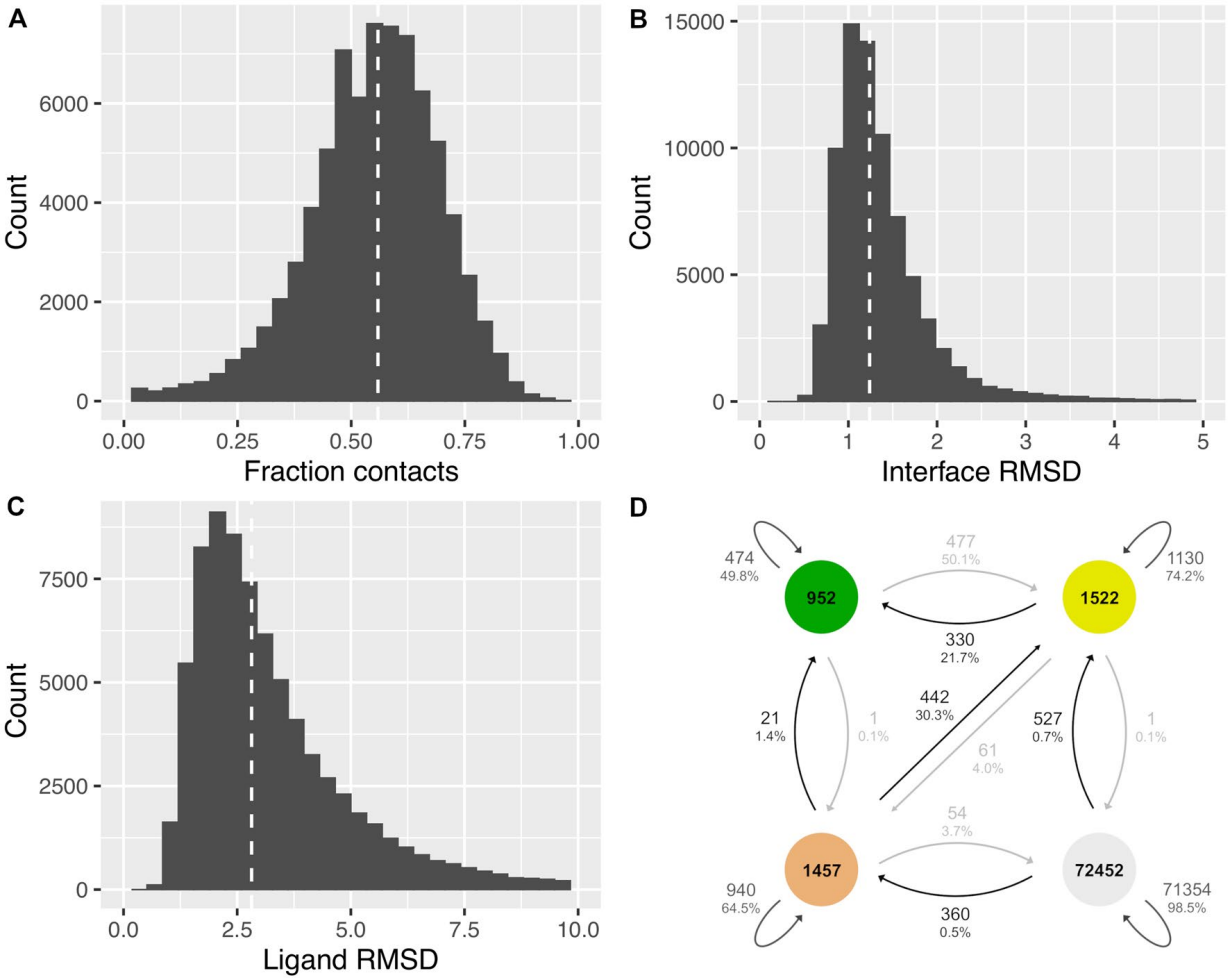
Quality assessment of the AlphaFold2-generated models in relation to its provided docking-generated template structure. Distribution in the (**A**) fraction of conserved template contacts, (**B**) interface RMSD and (**C**) ligand RMSD between the AF2-generated model and its provided docking-generated template for the decoys in the bound-backbone set. The RMSD calculations only include the Cɑ atoms. All decoys generated with ProPOSE, ZDOCK, PIPER and ClusPro were combined. The median of the distribution is indicated by the dashed white line. (**D**) Transitions from the docking-generated models to the corresponding AF2-generated models in terms of model quality relative to the crystal structure. Transitions between the high-quality and incorrect classes were removed for clarity. The transition from incorrect to high quality occurs in 212 instances (0.3%). Colors denote structure quality levels as defined by CAPRI classification: high (green), medium (yellow), acceptable (beige) and incorrect (grey). Figures in this paper were produced in R^45^ using the ggplot2 library^46^.

AlphaFold2 was run providing as input the amino-acid sequence and the structural template of each of the docking-generated model in the two sets. Noteworthy, only the backbone structure of the template was provided, i.e. the template was stripped of all its side-chains similarly with a previous study on protein folding^26^. This procedure allows the release of unnecessary constraints to the Structure module and forces a rebuilding of the side-chains by AF2 while allowing larger refinements to take place. We compared the AF2-generated model to its provided template to assess the amount of structural changes at the antibody-antigen interface that occur from rebuilding. Specifically, the fraction of conserved contacts and interface RMSD were calculated and used as proxy for assessing structural divergence (Fig. 1A, B). We found that 56% of the template contacts were retained upon remodeling. In terms of interface RMSD, the structures deviated by 1.24 Å. The deviations in interface RMSD propagate to the whole structure and lead to an overall rearrangement of the antibody by as much as 2.82 Å (Fig. 1C). These values were maintained in the unbound-backbone set (Fig. S1).

From the fraction of template and RMSD metrics, it remains unclear if such changes in interface contacts result in a loss or a gain of contacts relative to the ones observed in the experimentally-resolved structure. Therefore, we then compared the model quality of the docking-generated and AF2-generated structures to the experimental structures for the bound-backbone (Fig. 1D) and unbound-backbone (Fig. S1D) sets. In general, most models produced by AF2 retained the quality of their docking-generated templates. In absolute terms, there are more positives in AF2-generated models than docking-generated models, with 4974 and 362 positives as opposed to 3,931 and 291 positives, for the bound-backbone and unbound-backbone sets, respectively (Table 1).

One can note a relation between the ability of AF2 to increase model quality and the quality of the docking template used as input. High-quality docking models tend to lose the most of their quality, in part due to the stringency of the high-quality metrics paired with the inability of AF2 to reproduce physics at atomic level. These results underlined the inability of AF2 to accurately reproduce the fine atomic details at the interface from its lack of physics, as shown previously^10^. While applying AMBER minimization to the AF2-generated models might arguably have restored some of the native contacts at the antibody-antigen interface, salvaging a repacked interface normally falls outside the applicability range of standard minimization protocols that reach local optima in the energy landscape. In net numbers, there were 952 and 1037 positives of high-quality for docking-generated and AF2-generated models, respectively. Benefits of AF2 in producing better-quality models were seen mainly for docking templates of medium and acceptable qualities, with increases of 22% and 30%, respectively (Fig. 1D). These trends were maintained in the unbound-backbone set, with increases of 11% and 21%, respectively (Fig. S1D). It is encouraging to see improvements even for the models of acceptable quality, which are considered to be at the limit of practical applicability.

For clarity, while the terminology “AF2 rescoring” is often employed in this paper, “AF2 refinement” could be used interchangeably given the structural rearrangements incurred by this procedure. These structural changes pose a question on whether one should use the docking-generated or the AF2-generated models for comparisons to the crystal structures during success evaluation, as model selection could impact success levels. There is no definitive answer to this question. For calculating success rates, in this study we chose the AF2-generated models whenever AF2 rescoring was applied, and the docking-generated models when models were ranked by docking scores.

### AlphaFold2 rescoring improves true-positive ranking

**Figure 2:**
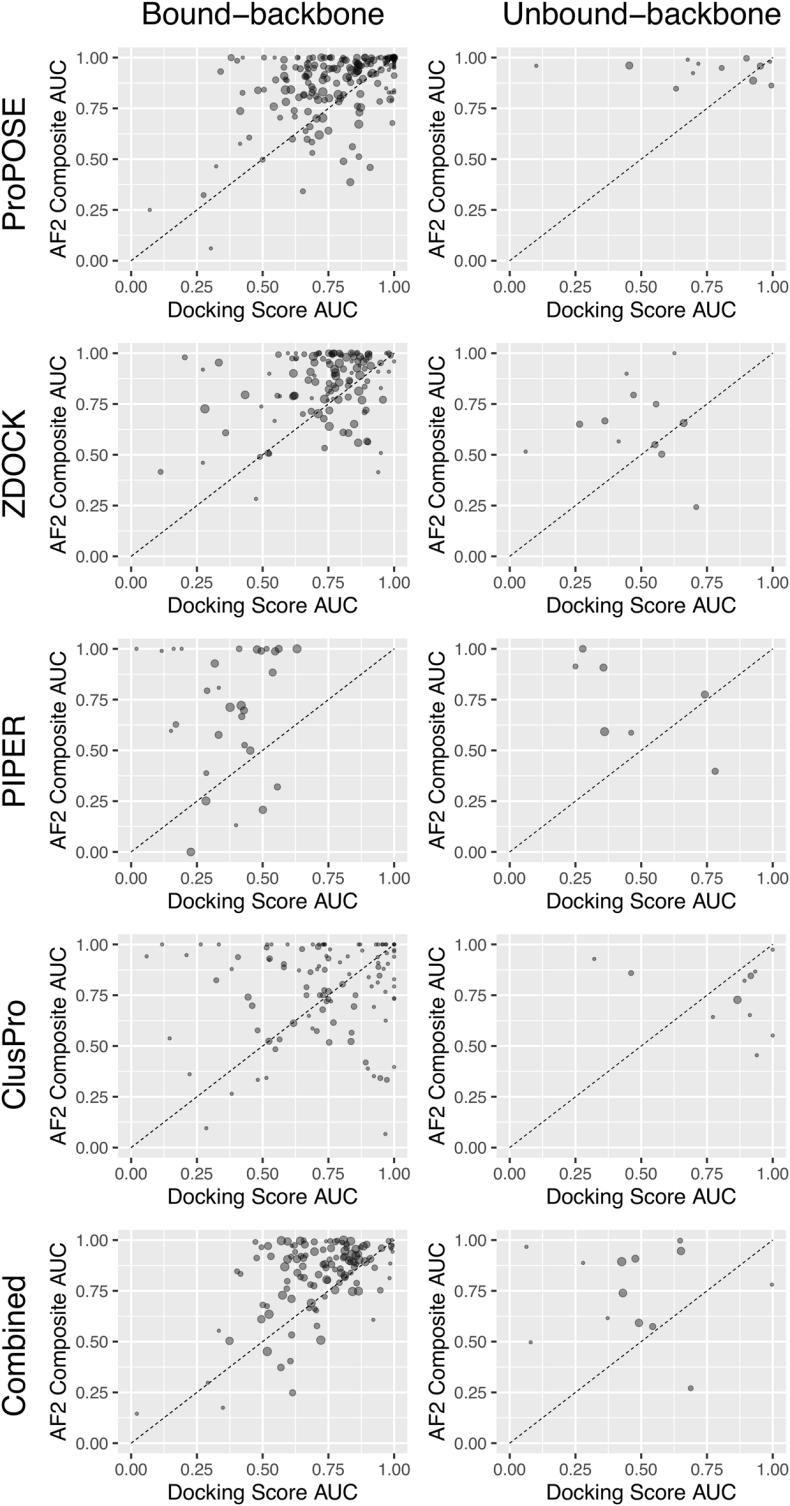
Classification of true positives and false positives according to AUC for the ROC curves based on docking scores relative to AF2 composite scores. Each point in the plot represents a different system. The points are area-weighted by the number of corresponding positives on a 1-to-10 scale. The diagonal line is drawn to indicate the impact of AF2-rescoring. The docking-generated (x-axis)and AF2-generated models (y-axis) were used for success evaluation relative to the corresponding crystal structure. Only those systems that had at least one true positive in both ranking schemes are plotted. The number of data points (antibody-antigen systems) in each plot are 206, 12, 129, 14, 87, 10, 107, 11, 216 and 20 (from left to right and top to bottom). AF2 rescoring improves the classification for 140 (68%), 9 (75%), 98 (76%), 10 (71%), 76 (87%), 9 (90%), 55 (51%), 2 (18%), 176 (81%) and 18 (90%) antibody-antigen systems. Positives include high, medium and acceptable quality models according to CAPRI classification^30^.

The area-under-the-curve (AUC) of the receiver-operating-characteristic (ROC) curve was calculated per each system to evaluate the overall classification of true positives and false positives. The AF2_Composite_ score was used in re-ranking antibody-antigen docking models. The AUCs of models ranked by docking scores were compared to the AUCs of models ranked by the AF2_Composite_ score for those systems with at least one positive model (Fig. 2). On average, the AUCs were significantly improved after rescoring with AF2_Composite_, and improvements were obtained for all three individual docking methods.

In the bound-backbone set, AF2_Composite_ improves the classification for 140 (68%), 98 (76%) and 76 (87%) systems having at least one positive model for ProPOSE, ZDOCK and PIPER, respectively. ProPOSE showed not much benefit from AF2 rescoring given its high accuracy in bound docking, hence an average AUC improvement of only 0.09 was obtained. Other docking methods benefitted more from the AF2 treatment, with average AUC improvements of 0.11 and 0.26 for ZDOCK and PIPER, respectively. ClusPro benefitted the least from a re-classification by AF2 with only 55 (51%) systems showing improvements in AUC, while a good portion of systems had a detrimental impact from AF2 rescoring. This result could potentially be explained by ClusPro and AF2 fulfilling similar roles with respect to the recovery of poses that were initially ranked lower. Nevertheless, AUC improvements of 0.05 were still observed from AF2 rescoring using ClusPro. When the acceptable-quality poses were removed from the definition of success (Fig. S2), AUC improvements increased for both ZDOCK (0.16) and PIPER (0.31) as noted by the higher density of points in the upper section of the plots, suggesting that poorer quality models were harder to score with high confidence by AF2. Still, it is impressive to see systems with poor or random AUC (< 0.6) based on docking scores improve to remarkable levels (AUC > 0.9) after AF2_Composite_ rescoring; these included 25 systems (33%) for PIPER and 13 systems (12%) for ClusPro.

In the unbound-backbone set, the classification is improved for 9 (75%), 10 (71%), 9 (90%) and 2 (18%) systems having at least one positive model for ProPOSE, ZDOCK, PIPER and ClusPro respectively (Fig. 2). The average AUC improvements in this set were larger than in the bound-backbone set, reaching 0.20, 0.20 and 0.22 for ProPOSE, ZDOCK and PIPER, respectively. When acceptable-quality models were removed, average AUC improvements were 0.11, 0.37 and 0.40, respectively. The AF2 rescoring was detrimental for ClusPro models on this set, with deteriorations of average AUC of 0.06 and 0.09 when retaining or not acceptable-quality models, respectively.

We also analyzed the combined set of decoys from all four methods (ProPOSE + ZDOCK + PIPER + ClusPro) to assess the ranking ability of AF2_Composite_ when challenged with models obtained from different physics-based methods which may slightly vary in their protein preparation. The AUCs were markedly improved after AF2 rescoring for 176 (81%) and 18 (90%) systems for the bound-backbone set and unbound-backbone set, respectively.

### AlphaFold2 rescoring improves early success rates

**Figure 3:**
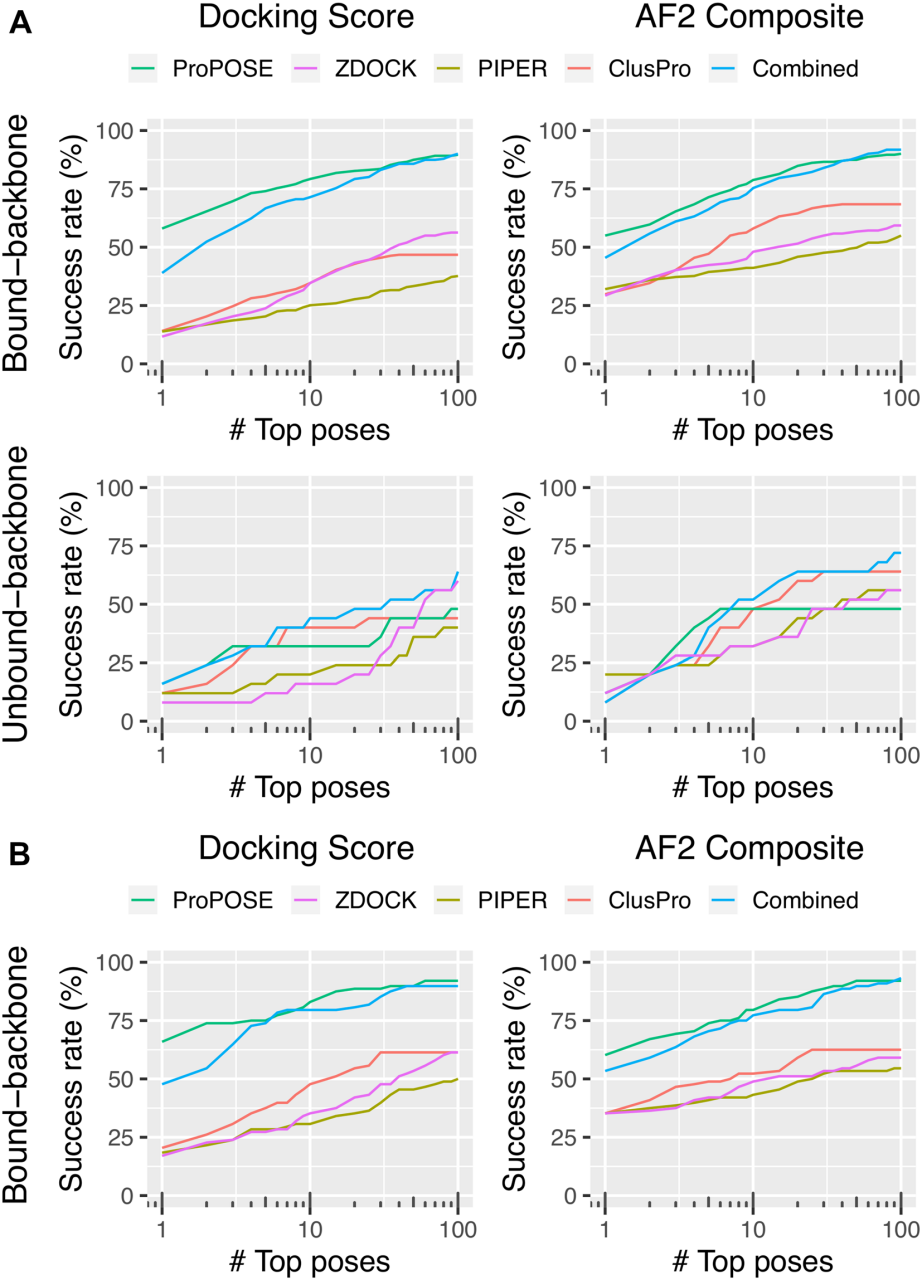
Success rates for various docking methods followed by AF2 rescoring as a function of the number of top-ranked models. The rates are shown for models ranked using the docking scores and after rescoring with the AF2_Composite_ score for the bound-backbone set and unbound-backbone set for systems that were released (**A**) before and (**B**) after the training of AlphaFold2. The docking-generated (left) and AF2-generated (right) models were used for success attribution relative to the corresponding crystal structure. An acceptable-quality model according to CAPRI classification^30^ was the minimum requirement for success.

The AUC is a global indicator of performance and is often insufficient to capture important early success in terms of true positives enrichment. Having better AUC from rescoring should normally translate into higher success rate in the top fractions, but it remains unclear how many poses have to be inspected by the user to get one successful model. The AUC also does not capture the systems in which docking failed by producing only negatives in the top-100 models while AF2 succeeded in rescuing some of those as positives. For these reasons, success rates were calculated along the ranked list of models and plotted on a logarithmic scale (Fig. 3). We distinguish late success rates (top 100) from early rates (top 1 and top 5). Here, the top-5 success rates were used as it is generally a tractable number of models to inspect. The success rates based on docking scores and docking-generated models were again compared to those obtained after re-ranking with the AF2_Composite_ score and AF2-generated models. The success rates at key metrics (top-1, top-5 and top-10 ranked models) were also reported and tabulated as a function of model quality (Tables 2, S1 and S2).

**Table 2:** Success rate comparison between docking-generated-template and template-free modeling of antibody-antigen complexes with acceptable or higher quality (CAPRI).

Method	Top-1		Top-5		Top-10	
	Standard scoring (%)	AF2 re-scoring (%)	Standard scoring (%)	AF2 re-scoring (%)	Standard scoring (%)	AF2 re-scoring (%)
Bound-backbone (in AF2 training set)						
ProPOSE	58	55	74	71	79	79
ZDOCK	12	29	24	42	35	48
PIPER	14	32	20	39	25	41
ClusPro	14	30	29	47	35	58
Combined	39	45	67	66	71	75
AF2-Multimer	N/C^a^	N/A^b^	N/C	N/A	N/C	N/A
Unbound-backbone (in AF2 training set)						
ProPOSE	16	12	32	44	32	48
ZDOCK	8	12	12	28	16	32
PIPER	12	20	16	24	20	32
ClusPro	12	8	32	32	40	48
Combined	16	8	32	40	44	52
AF2-Multimer	N/C	N/A	N/C	N/A	N/C	N/A
Bound-backbone (in AF2 test set)						
ProPOSE	66	62	75	75	84	80
ZDOCK	18	35	27	42	35	48
PIPER	18	36	29	42	32	44
ClusPro	19	35	38	49	48	53
Combined	49	55	75	71	80	78
AF2-Multimer	22	N/A	28	N/A	N/M^c^	N/A

^a^N/C: not calculated due to bias in assessing AF2 performance based on structures present during its development phase.

^b^N/A: not applicable.

^c^N/M: not calculated due to 5-models limit of AF2-Multimer.

In the bound-backbone set, the success rates plateaued near the top-50 mark with late success at 90%, 56% and 38% for ProPOSE, ZDOCK and PIPER, respectively (Fig. 3A). ClusPro plateaued near the top-30 mark at 47% given its lower number of predicted models after clustering. AF2 rescoring showed little to no benefit on late success rates for ProPOSE and ZDOCK with 90% and 59% but showed significant benefit for PIPER and ClusPro with rates up to 55% and 68%. The success rates were further broken down by model quality (Figs. S3 and S4), and indicated a deterioration in model quality for ProPOSE after AF2 refinement, and improvements in model quality especially in the top fractions for ZDOCK, PIPER and ClusPro after AF2 refinement. AF2 rescoring also helped with the early success of recovering true positives. While the early success for ProPOSE remained unchanged after AF2 rescoring, the rates at the top-1 model level for ZDOCK, PIPER and ClusPro were markedly improved to reach 29%, 32% and 30% from 12%, 14% and 14%, respectively. At the level of top-5 models, success rates for ZDOCK, PIPER and ClusPro reached respectively 42%, 39% and 47% after AF2 rescoring from 24%, 20% and 29% based on docking scores. Overall, it is reassuring to see that AF2 enhanced antibody-antigen docking predictability in this more trivial but still important control experiment, i.e., bound-backbone docking. Also, it is reassuring to notice improvements even for models that went through further processing with ClusPro, which corroborate previously results obtained for generic protein–protein interactions^25^. It is reasonable to assume that similar conclusions might have been obtained on IRAD-reranked models^28^ in the case of ZDOCK. Improvements in success rates are always more important going from top-1 to top-5 models than going from top-5 to top-10 models, with ClusPro benefitting the most out of top-10 solutions.

As a side note, the use of a 200-residue indexing gap has been proposed as an alternate strategy over the use of artificial linkers for joining protein chains^31^. While the gap indexing strategy generated predicted structures with higher overall confidence values over the linker strategy, in our hands it had very little to no impact on early success rates (Fig. S5), as others have also previously reported^19^. The gap indexing methodology appears to be preferred for late success rates according to our data.

In the unbound-backbone set, the trends were nearly identical with those seen in the bound-docking experiment (Fig. 3A). The AF2 rescoring showed no benefit for ProPOSE and ZDOCK with regards to late success rates, but it showed benefits for PIPER and ClusPro with late success rates going up to 56% and 64%, from 40 and 44%, respectively. While success rates with the top-1 model remained generally unchanged after AF2 rescoring (only PIPER showing some improvements), the top-5 models afforded superior success rates for the methods employed with the exception of ClusPro. Hence, success rates of 32%, 12% and 16% were obtained in the top-5 models for ProPOSE, ZDOCK and PIPER, respectively, when ranked with their internal scoring functions. Upon rescoring with the AF2_Composite_ score, these rates went up to 44%, 28% and 24%, respectively. Another way to see this improvement is that a late success rate of 48% reached at the top-90 level with the ProPOSE scoring function was achieved much earlier, at top-6 models only, after AF2 rescoring. According to top-10 predictions, both ProPOSE and ClusPro predict equal amounts of complexes with a larger proportion of higher quality models for ProPOSE (Tables 2, S1 and S2).

Pulling together the models from all four methods followed by AF2 rescoring achieved a success rate of 40% and 72% in the top-5 and top-100 models for unbound docking. Despite differences in size and composition of the datasets, these success rates are higher than what was ever achieved in antibody-antigen unbound docking thus far, which neared 20% and 35% at the top-10 level for ZDOCK and ClusPro, respectively^32^. Further analysis showed that medium-quality models were preferentially enriched over acceptable-quality models upon rescoring with AF2, reinforcing the notion that poorer quality models are harder to score with high confidence by AF2 (Fig. S4).

One could argue that the improvements obtained after AF2 rescoring may have been somewhat biased by the AF2 model potentially “remembering” the antibody-antigen systems examined here as being part of the very large AF2 training data set. As control for this potential bias, we assembled a separate bound-backbone set and tested it. This control set included 88 recently solved structures of antibody-antigen complexes disclosed after the training of the AF2 model (see Methods section). The results obtained for this recent dataset showed that late success rates were nearly identical across the panel of methods evaluated while the trends in early success rates also remained consistent with the earlier dataset (Fig. 3B). In this recent dataset, the AF2 rescoring was beneficial for early success rates with improvements at top-1 level going up to 35%, 36% and 35% from 18%, 18% and 19% for ZDOCK, PIPER and ClusPro, respectively. When the top-5 models were considered, these success rates were improved to 42%, 42% and 49% from 27%, 29% and 38%. To draw a comparison to template-free modeling, the performance of AF2-Multimer against this set was 22% and 28% in the top-1 and top-5 models, respectively. This is considerably higher than the 20% in the top-5 previously reported using an older version of AF2-Multimer^21^. Hence, these results demonstrate that the performance of template-based modeling exceeds the one of template-free modeling, with the caveat of the larger computational cost.

### AlphaFold2 composite score can separate positives from negatives

**Figure 4:**
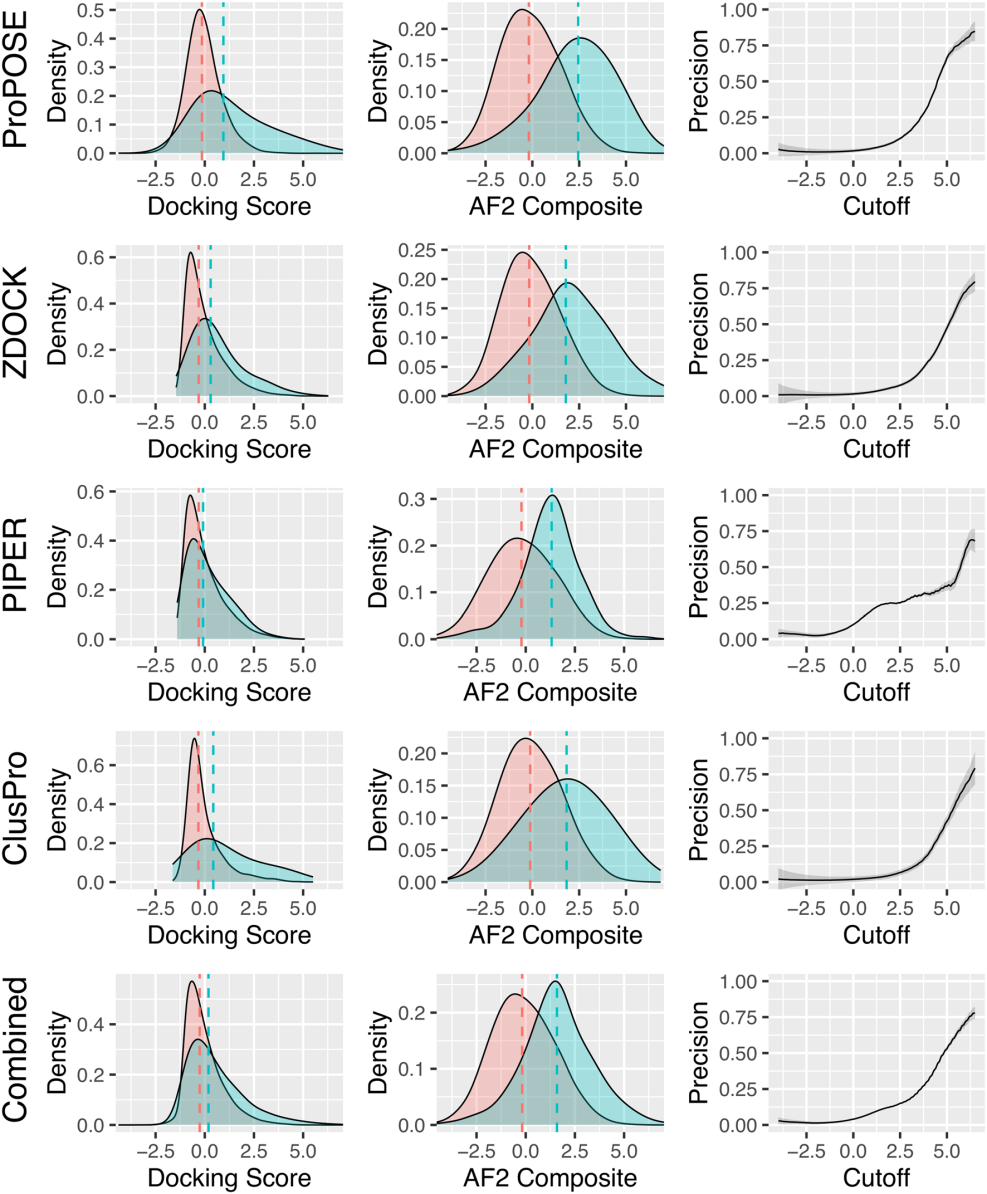
Density plots for the docking scores and the AF2_Composite_ score for the negative and positive sets from the bound-backbone set. The means of the distributions are marked with dashed lines. Precision curves were built from calculating the fraction in the number of true positives over the total number of true positives and false positives according to a given cutoff in the AF2_Composite_ score. The smoothed densities were used to build the precision curves to avoid outlier bias. Shadings around the precision curves indicate the errors on these curves, which were estimated based on the number of data points, i.e. less data points incur larger errors. For all docking methods evaluated, the likelihood of a true positive event increases with higher cutoff values in the AF2_Composite_ score.

The density distribution of scores for the positives and negatives obtained with the docking methods covered in this study and with the AF2_Composite_ function were comparatively analyzed. For the bound-backbone set, the separation between the medians of the distributions of positives and negatives were 1.1, 0.6, 0.2 and 0.8 standard deviations based on the normalized docking scores from ProPOSE, ZDOCK, PIPER and ClusPro, respectively (Fig. 4). Thus, the ProPOSE score appears to already discriminate to some extent the true positives from decoy models in the bound-docking scenario. Rescoring with the AF2_Composite_ score increased the separation between positives and negatives for all three methods to 2.6, 2.0, 1.5 and 1.9 standard deviations. This highlights a stronger overall power in discriminating true positives with the AF2_Composite_ scoring scheme that is less subjective to small perturbations in the structure. The differences in separations achieved with the AF2-reranked models between the various docking methods (PIPER < ClusPro < ZDOCK < ProPOSE) also highlight that docked decoy sets should not be treated equally and suggest an influence of model quality on the achieved AF2 confidence levels.

One important property of a metric is its ability to set a threshold from which one can draw confidence levels of success and obtain more valid conclusions. This has implications in knowing, for instance, if a prospective docking run succeeded in generating top-ranked near-native poses or not. To this end, precision curves were built from the smoothed probabilities for positives versus negatives as a function of a sliding threshold for the AF2_Composite_ score (Fig. 4). These precision curves describe the ratio of true positives found over the number of models sampled at a given threshold of the score. The precision curves are consistent across the decoy sets generated with the different docking methods. For example, a cutoff set at a value of 5.0 for the AF2_Composite_ score suggests a 70% chance of successfully generating a native-like antibody-antigen model when using ProPOSE for docking.

Similar trends were obtained for densities and precision curves with the unbound-backbone set (Fig. S6). The strong benefit of AF2 rescoring is highlighted by improvements from 0.9, 0.0 and 0.2 to 3.2, 1.1 and 1.1 standard deviations for ProPOSE, ZDOCK and PIPER, respectively, in separating positives from negatives. The case of ClusPro is peculiar as a detrimental impact after AF2 rescoring is observed, from 1.4 to 1.2 standard deviations. Notably, ClusPro produces the largest amount of acceptable-quality models on this set (Fig. S4). However, the precision curves are plagued by the low counts of systems in this set. The calculated errors are much higher provided the weaker statistics. If more systems and better-quality structures closer to the native complex could be fed into AF2, most likely better precision curves that approach those seen in the bound-backbone set could be obtained in unbound docking as well.

## Discussion

### AlphaFold2 helps alleviate the scoring problem

In this study, we hypothesized that AlphaFold2 could be used to circumvent the weakness of physics-based docking methods with regards to scoring antibody-antigen complexes. A first assumption was that docking methods, based on their stronger underlying physics, are generally well-suited for finding plausible binding modes for antibody-antigen complexes. A second assumption was that docking scores are generally unable to distinguish true positives from false positives. In short, it was overall assumed that docking methods can be used for the purpose of sampling but not scoring. The AF2_Composite_ score was derived to perform the task of scoring and re-assess the acceptability of the docking-generated models.

The AF2_Composite_ score disregards the predicted energy of the physics-based methods and is solely based on the pLDDT and pTMscore confidence metrics from AF2. These two metrics capture distinct and complementary features for compounding the structural errors. On the one hand, pLDDT is a more relevant metric for local error approximation. On the other hand, pTMscore is a global metric better suited for larger systems such as in the prediction of complexes^10^. The normalized values for the pLDDT and pTMscore scores were summed to derive the AF2_Composite_ score. The use of normalized scores usually implies a requirement for normal distribution of data. While the distributions of AF2_Composite_ scores for the individual systems are not perfect, they do not deviate much from normality (Fig. S7). We found this summation to be central for success. In fact, using either metric in isolation led to significant lower performance (Fig. S8). The AF2_Composite_ detects outstanding models for which the normalized pLDDT and pTMscore metrics agree on high scoring and favorable ranking with high confidence. This agreement is a strong determinant for describing the quality of the model (Fig. S9). We noted that this agreement weakens as the quality of the docked model deteriorates. For example, pLDDT and pTMscore cross-correlate poorly (R^2^ < 0.4) on models of acceptable quality alone, whereas higher correlations are seen on high and medium-quality models. Our results showing improved classification after rescoring also indirectly point to the same conclusion, that is, that these metrics agree better for positives than negatives (Fig. 2). The AF2_Composite_ score is a convenient, simple yet effective approach for scoring that is not subjective to any sort of training or calibration.

It is difficult to compare the absolute values for the pLDDT and pTMscore metrics across various protein systems and docking methods, as they are influenced by many factors such as protein preparation, format and input structure. Therefore, the components of the AF2_Composite_ score were standardized, which makes the AF2_Composite_ score system-independent. One drawback of data normalization is the inability to discriminate success; a distribution comprised only of negatives may look identical to one of only positives. However, an important aspect of the AF2_Composite_ is that its distribution is no longer a standardized normal distribution, due to its reliance upon the agreement between its component terms. Incorrectly predicted models have lower agreement in their pLDDT and pTMscore metrics (Fig. S9) and lower values of those metrics in absolute terms (Fig. S10). Inversely, medium and high-quality models agree more on their confidence metrics while having higher values in absolute terms. Statistically, an initial distribution composed only of incorrectly predicted poses would suddenly appear skewed as higher quality models are introduced in it. The AF2_Composite_ score can thus be treated in absolute terms and allows the definition of a threshold that can be used to discriminate the docking runs that succeeded from those that failed (Fig. 4).

### Structure quality of the template matters

Although for some complexes we observed a significant remodeling of the antibody-antigen interface leading to higher-quality models or success, it is not guaranteed that this is a general behavior. In fact, for most complexes, the AF2 remodeled structure retained the quality of the supplied docked model (Fig. 1). In this study, we used a quite loose definition for success that includes acceptable quality by CAPRI metrics, which is standard practice the field^30,33^. However, model quality has a major influence on the confidence scores. Not only the high and medium-quality models have more value because of higher agreement of normalized pLDDT and pTMscore components, their absolute confidence scores approach those of crystal structures (Fig. S10). Many systems had binding interfaces identified only broadly (acceptable-quality), which is not always sufficient as those models were predicted with much less confidence.

Given the importance of model quality for high-confidence scores, the docking methods that tend to generate larger proportions of high and medium-quality models should be preferred for rescoring by AF2 instead of those that produce a large fraction of acceptable-quality models. The unbound docking with ProPOSE is one such case which benefitted most from AF2 rescoring and led to the most impressive AUC (Fig. 2) and early success rate results (Fig. 3). The improvements incurred from AF2 rescoring of ZDOCK and PIPER poses are less striking in that regard. Along these lines, ClusPro processing of PIPER docking poses may also be less suited for AF2 rescoring, since in this study along with a previous comparative study ClusPro produced the largest number of acceptable-quality models among all docking methods evaluated^34^.

### Influential factors for AlphaFold2 success

The first of several decisions made in this study was the granularity of protein sequence used by AF2 onto the docked template structure, which turned out to be of critical importance. We found that providing a full atomistic template with side-chains added too many constraints to AF2. It prohibited the Structure module of AF2 to work with its own scoring scheme and instead forced to work in the context of another scoring function. Using the full side-chain atomistic model led to significantly worse performance (Fig. S11). Conversely, forcing the protein sequence of the template to poly-alanine allowed freedom to the AF2 Structure module to remodel the antibody-antigen interface with internally consistent structural determinants for binding, which were necessary for assessing model confidence accurately. Moreover, by allowing AF2 to remodel the binding interface, we simplified the docking task to finding an overall good docking pose among several given backbone geometries. This is an easier problem than having to predict the fine atomic details at the interface, given the size of the search space to be explored.

Secondly, a choice in the final structural models used for AF2 rescoring had to be made. The side-chain reconstructed models produced by AF2 were noted to be different structurally than the templates at fine atomic levels (Fig. 1). In some cases, these changes were sufficiently large enough to be attributed a different quality, or even different arrangements at the backbone level that could qualify as local "redocking". A few illustrative examples of possible structural rearrangements of the docking poses obtained upon reconstruction with AF2 are shown in Fig. S12. One decision that had to be made was which template to use for evaluating success. The overall net sum of positive models was greater for the AF2-reconstructed models when compared to the initial docking-generated ones. Therefore, throughout the study, we hence used the models reconstructed by AlphaFold2 and thereby chose to accept the quality of its structural refinement. On that aspect, our study fundamentally differs from the one of Roney et al*.* in which the authors weighted their composite score by the TMscore, which was required for appropriately scoring the templates. By penalizing structural deviations of the AF2-generated model relative to its template, they ensured the confidence metrics of AlphaFold favor the templates over the models. Applying a similar weighting scheme to emulate this agreement in structure showed no benefit on our data (Fig. S8). However, a decrease in the number of high-quality structures was noticed for the bound-backbone set due to the less atomistic physical method in AF2. One suggestion for practical use is that the choice of the template should be based on the application. In applications that require clash-free structures with finer atomic details such as affinity maturation, one should employ docking-generated models. For coarser applications like epitope mapping, the AF2-reconstructed models are preferred according to our results here.

Another decision made was related to the number of docking models per system. The AF2_Composite_ score is inevitably influenced by the number of models included per system given that its component terms are normalized (Z-scores). Higher number of models are required to obtain a better approximation for the distribution and derive more accurate composite scores (Fig. S13). For instance, standardized values obtained from a distribution with only 5 models are more likely to have larger errors than on one obtained from 100 models. Including the top 25 poses appears to be sufficient to reduce the error below one unit of AF2_Composite_ score. Therefore, generating more docking models not only leads to an increase likelihood of visiting near-native poses, but also to more accurate confidence scores. Improvement in success would most likely be observed if the top-1000 docking models could be included. However, large number of models per system come with an increased computational cost. If one cannot afford hundreds of AF2 calculations per system, resampling techniques such as bootstrapping^35^ should be considered.

One common practice in the field consists in combining many methods for consensus prediction in order to circumvent the limitations and inherent biases from training each method^36,37^. Docking methods are no different on that regard, with some dockers being more successful on specific protein systems. By combining multiple methods like ProPOSE, ZDOCK, PIPER and ClusPro, we hoped to also benefit from their orthogonality and increase success rates. While there were significant improvements in global terms (e.g., AUC-ROC), early success rates after AF2 rescoring improved only marginally when all docking methods were combined relative to individual docking methods. Lastly, each physics-based scoring function has specific biases, approximations and constraints in their underlying force-fields, which lead to varying sensitivities to fine structural details. Scoring effectively and fairly models produced from a variety of docking methods is a difficult task in consensus modelling. By using poly-alanine docking templates, we showed in this study that AF2 can be used as an effective platform for rescoring complexes in an unconstrained environment irrespective of the physics-based docking method and without a need for prior training. This is a promising finding for future method developments.

## Methods

### Antibody-antigen systems

The antibody-antigen complexes used in the present study are from the union of the protein–protein benchmark version 5.0 dataset^38^ and the antibody benchmark dataset^4^ originally collected from SAbDab^39^. This led to a set of 231 complexes, which we call here the “bound-backbone” set. For 25 antibody-antigen systems from this set, we also found crystal structures of the unbound partners from the same benchmark^38^, and these structures were collected into the so-called “unbound-backbone” set. All complexes from the “bound-backbone” and “unbound-backbone” sets were part of the training data of AlphaFold2 which could potentially bias the results. To provide a more objective view of the impact of AF2 rescoring, we assembled an additional “bound-backbone” set composed of 88 antibody-antigen systems published from January 1^st^, 2022 (after the cut-off date for the training of AlphaFold version 2.3), which we tested and analyzed separately from the other sets.

The side-chains of the molecules in the bound-backbone sets were repacked using SCWRL4^40^ after complex dissociation. For each of the bound-backbone and unbound-backbone sets, we generated 100 docking models per system with each of the three docking methods described below. Symmetric units were detected by sequence alignments and assembled using PyMOL^41^.

### Docking methods

ProPOSE^4^ distributed version 1.0.2, ZDOCK^2^ version 3.0.2 and PIPER^3^ version 0.0.4 were employed to perform the docking simulations. Initially, all input molecules were repaired (addition of missing side-chains), prepared (removal of small molecules, addition of hydrogen atoms, addition of capping groups where needed) and then charged and energy-minimized using the Amber force-field^42^ and suite of tools^43^. The minimization protocol applied is identical to the one previously described^4^ that retains the original backbone geometry of the input structure. Hydrogen atoms and capping groups were removed prior to performing docking with ZDOCK and PIPER for incompatibility reasons. ClusPro^34^ was used to post-process the top 1,000 predicted models generated by PIPER using only interface Cα atoms with a distance threshold between clusters of 9.0 Å. For PIPER and ClusPro, antibody-antigen atom parameters versions 0.0.4 and 0.0.6 were used.

Docking was directed to the complementarity-determining region (CDR) of each antibody. The CDR boundaries were defined using the Kabat numbering scheme^44^. Restriction to the CDR region was enforced for each of the docking methods as the following. For ProPOSE, the atoms within the CDR region were labeled with the HITSET flag. For ZDOCK, all atoms outside the CDR region were forced to have the atom type 19. For PIPER, masking spheres of 1 Å radius were applied onto all non-CDR atoms. No restriction was provided on the contact surface of the antigen.

### AlphaFold2 preparations

The multiple chains from the antibody-antigen docked models were connected into single chains by using 50-residue-long artificial linkers one-hot encoded as unknown residues in the final sequence and not modeled by AF2. The residues of the merged chains were renumbered in sequential order causing both ends of unsolved loops to be artificially joined, which may potentially have impacted the confidence values and predictability of AF2. The merged 3D structure was used as template for AF2 modeling. Hence, AF2 was provided as input the docked 3D template and the concatenated single-chain or “monomer” sequence for extraction of features data. The *template_all_atom_masks* input feature was used to apply an inclusive mask to the backbone and Cβ atoms to indirectly strip all side-chains from the template structure and artificially mutate all amino acids to alanine. Glycine residues were added the missing Cβ atoms. The *template_sequence* and *template_aatype* features are the alanine sequence and one-hot encoded alanine sequence of the template, respectively. The *template_domain_names* feature was defined as none. The multiple sequence alignment data used to build the monomer features were blanked and only contained the full sequence of the merged chains.

AlphaFold version 2.2.2 was used throughout this study for producing all data of the figures. The model model_1_ptm was used while all other AF2 parameters were kept to the default values, i.e. num_recycle 3 with stop tolerance 0.0. No subsequent minimization with AMBER was applied to the AF2-generated models. AlphaFold version 2.3.1 was used exclusively for comparison to template-free modeling and was run through ColabFold version 1.5.2 using the five model_*_multimer_v3 models. ColabFold was run with default parameters, i.e. unpaired-paired MSA and num_recycle 20 with stop tolerance 0.5. For consistency with the AlphaFold version 2.2.2 protocol, no AMBER minimization was applied. ColabFold was provided with the complete sequence of the complex, i.e. one that does not lack unsolved loop regions. The input sequences are provided as part of the Supplementary Information.

The AF2 calculations were run on NVidia A100 Ampere, P100 Pascal and V100 Volta cards on the Compute Canada clusters. The ColabFold calculations were run on GeForce GTX 1080 Ti and RTX 2080 Ti on the local computing clusters at the National Research Council Canada. The runtimes for running AF2 on the Compute Canada clusters are provided in the Supplementary Information (Fig. S14).

### Success evaluation

Success was evaluated by a comparison of models to the crystal structure using a combination of: (i) the fraction of native contacts, which is the fraction of antibody-antigen contacts observed in the crystal within an inter-atomic distance of 5 Å and preserved in the predicted model, (ii) the interface RMSD, which is the root-mean-square deviation in the set of antibody-antigen interface backbone atoms in the crystal according within an inter-atomic distance of 10 Å after the best-fit superposition, and (iii) the ligand RMSD, which is the root-mean-square deviation in antibody backbone atoms after the best-fit superposition of the antigen. The three metrics were combined for quality assignment as described previously^30^.

### Scoring schemes

From the antibody-antigen models predicted by AF2, confidence metrics were extracted at every position in the sequence. The scores pLDDT and pTMscore average the confidence metrics over the entire antibody-antigen models and report the confidence score as a singular value. The value typically falls within the range 0 (not probable) to 1 (highly probable). These scores were used to derive the AF2 composite score used in ranking and defined according to Eq. 1.

1 $$AF2_{Composite} = z_{pLDDT} + z_{pTMscore}$$

The *z*_pTMscore_ and *z*_pLDDT_ are standardized scores (or Z-scores) that are calculated from an ensemble of docked models for one given system. The Z-scores were not weighted to avoid any potential overfit and to allow an increased transferability between systems. Higher AF2_Composite_ scores are indicative of higher confidence relative to the ensemble.

The energetic scores outputted by ProPOSE, ZDOCK and PIPER were also collected and used in ranking the models. For ClusPro, the scores were equivalent to the size of the clusters. For clusters of same size, we prioritized clusters in the rank-order as they appear in the ClusPro output. To be consistent with the values reported by AF2_Composite_, for ProPOSE and PIPER, the sign of the scores were flipped such that higher scores are more favorable energetically. The scores outputted by docking methods cannot be compared in absolute units across docking methods. With the objective to compare and eventually aggregate docking poses and scores obtained from various docking methods, a standardization scheme was also applied to the energetic scores calculated in the ProPOSE, ZDOCK, PIPER and ClusPro docking runs. While the standardization is flawed as it assumes normal distributions and that docking methods produce similar distribution of scores, we believe it is a good compromise and is how one would go about merging docking methods.

### Supplementary Information

Supplementary Information 1.

Supplementary Information 2.

Supplementary Information 3.

## Data Availability

All data generated or analyzed during this study are included in this published article and its supplementary information files.
