# Supplementary material for: Enhanced antibody-antigen structure prediction from molecular docking using AlphaFold2
Source: Sci Rep. 2023 Sep 13;13:15107. doi: 10.1038/s41598-023-42090-5 (PMC10499836; doi:10.1038/s41598-023-42090-5)
Supplement: Supplementary file 1 — Supplementary Information 1. [file 41598_2023_42090_MOESM1_ESM.pdf]

Supplementary Information

for

Enhanced antibody-antigen structure prediction from molecular docking using AlphaFold2

Francis Gaudreault[1], Christopher R. Corbeil[1] and Traian Sulea[1,2*]

[1] Human Health Therapeutics, National Research Council Canada, 6100 Royalmount Avenue, Montreal, QC, H4P 2R2, Canada

[2] Institute of Parasitology, McGill University, 21111 Lakeshore Road, Sainte-Anne-de-Bellevue, QC, H9X 3V9, Canada

[*] Corresponding author: traian.sulea@nrc-cnrc.gc.ca

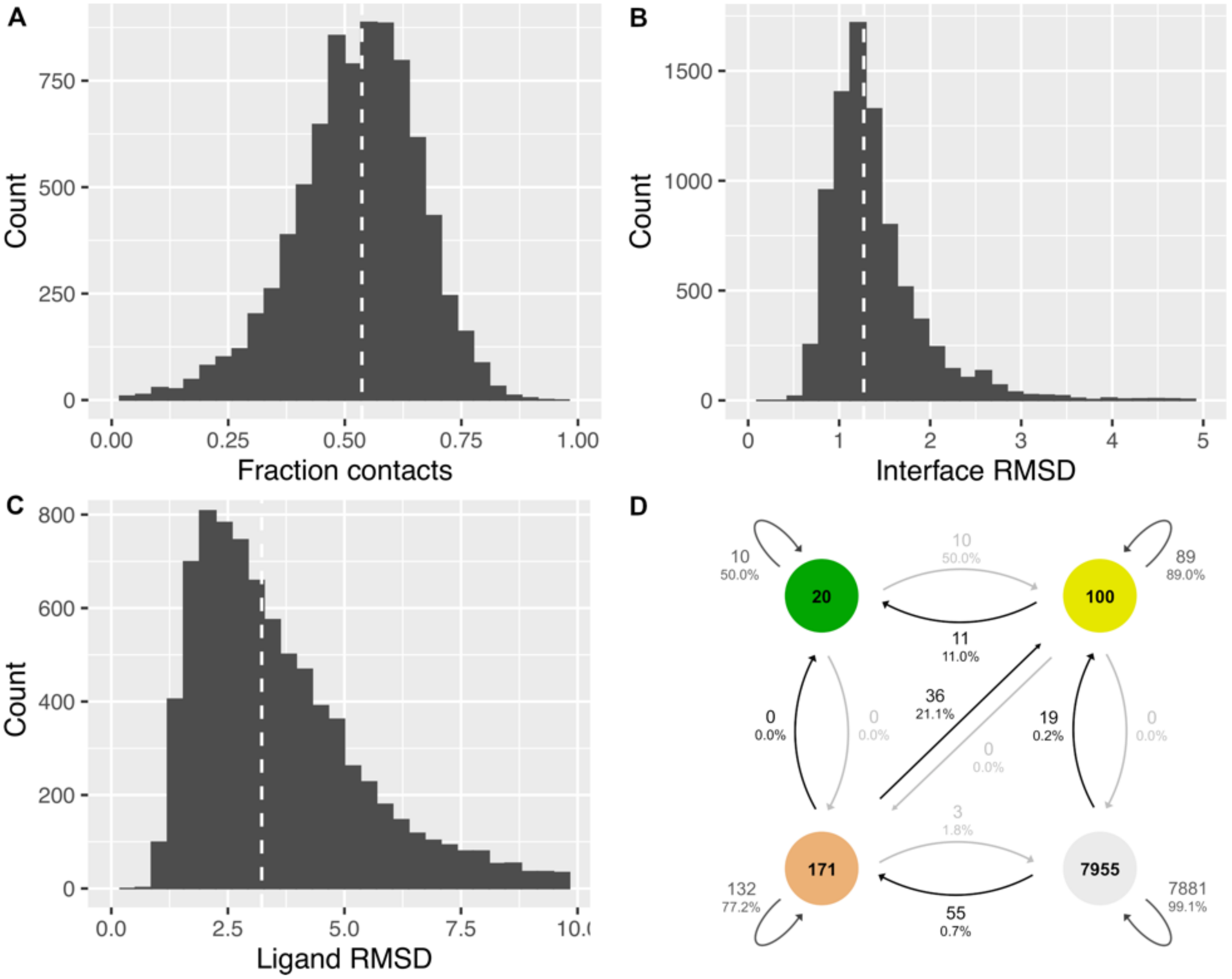

Figure S1. Quality assessment of the AlphaFold2-generated models in relation to its provided docking-generated template structure. Distribution in the (**A**) fraction of conserved template contacts, (**B**) interface RMSD and (**C**) ligand RMSD between the AF2-generated model and its provided docking-generated template for the decoys in the unbound-backbone set. The RMSD calculations only include the Cα atoms. All decoys generated with ProPOSE, ZDOCK, PIPER and ClusPro were combined. The median of the distribution is indicated by the dashed white line with values 0.54, 1.27 and 3.22. (**D**) Transitions from the docking-generated models to the corresponding AF2-generated models in terms of model quality relative to the crystal structure. Transitions between the high-quality and incorrect classes were not observed. Colors denote structure quality levels as defined by CAPRI classification (see Methods): high (green), medium (yellow), acceptable (beige) and incorrect (grey).

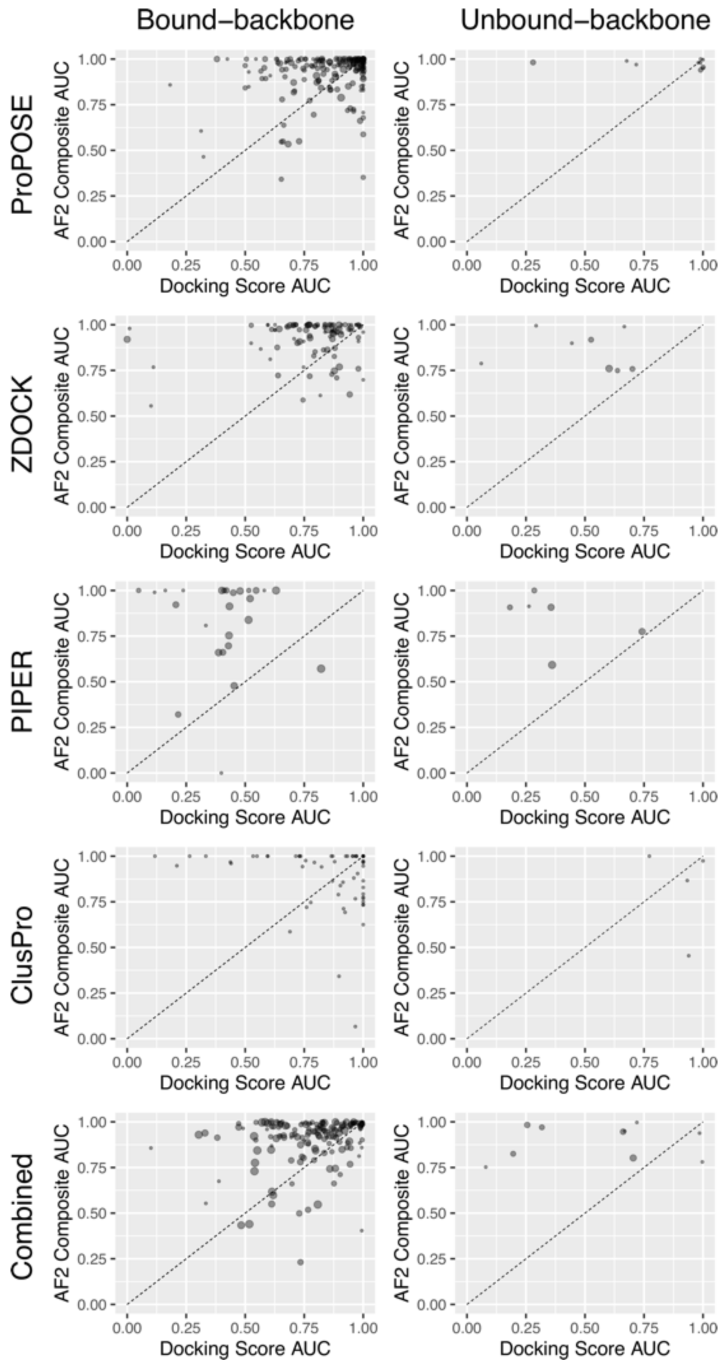

Figure S2. Classification of true positives and false positives according to AUC calculated from the docking scores compared to AF2-rescored for the bound-backbone and unbound-backbone sets. A medium-quality model is required for establishing success as opposed to an acceptable-quality (**Figure 2**). The docking-generated (left) and AF2-generated (right) models were used for success attribution relative to the corresponding crystal structure. Only those systems that have at least one true positive in both ranking schemes are plotted. The number of data points in each plot are 197, 10, 99, 8, 71, 8, 61, 4, 207 and 15 (from left to right and top to bottom). AlphaFold2 improves the classification for 115 (58%), 4 (40%), 84 (85%), 8 (100%), 64 (90%), 8 (100%), 28 (46%), 1 (25%), 168 (81%) and 13 (87%).

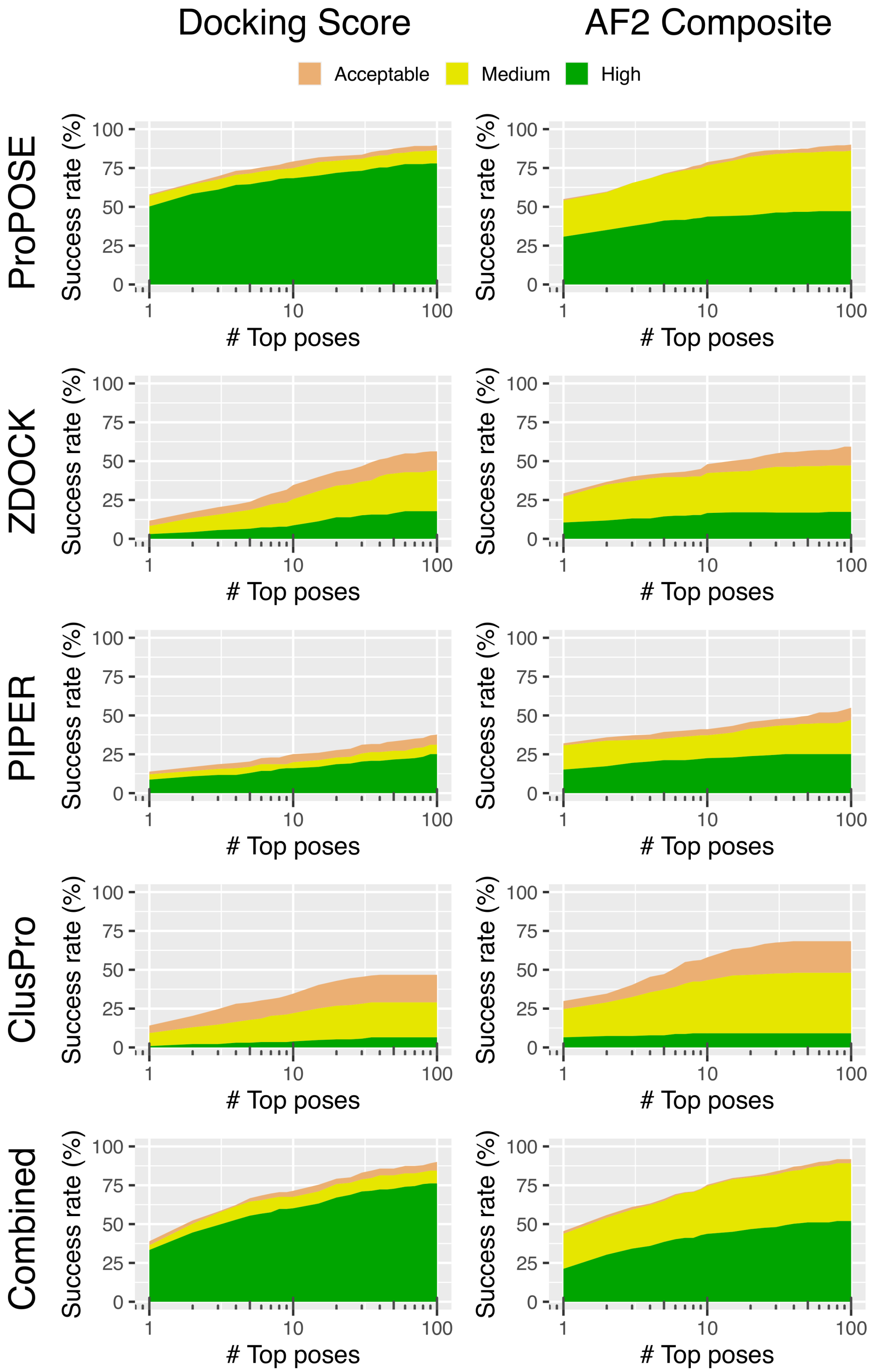

Figure S3. Success rates broken down for each model quality (see Methods) for the docking methods as a function of the number of top models considered from the ranked-list. The rates are shown for models ranked using the docking scores and the $AF2_{Composite}$ score for the bound-backbone set. The rates were plotted on a logarithmic scale for better visibility. The success was evaluated using the docking-generated (left) and AF2-generated (right) models relative to the corresponding crystal structure.

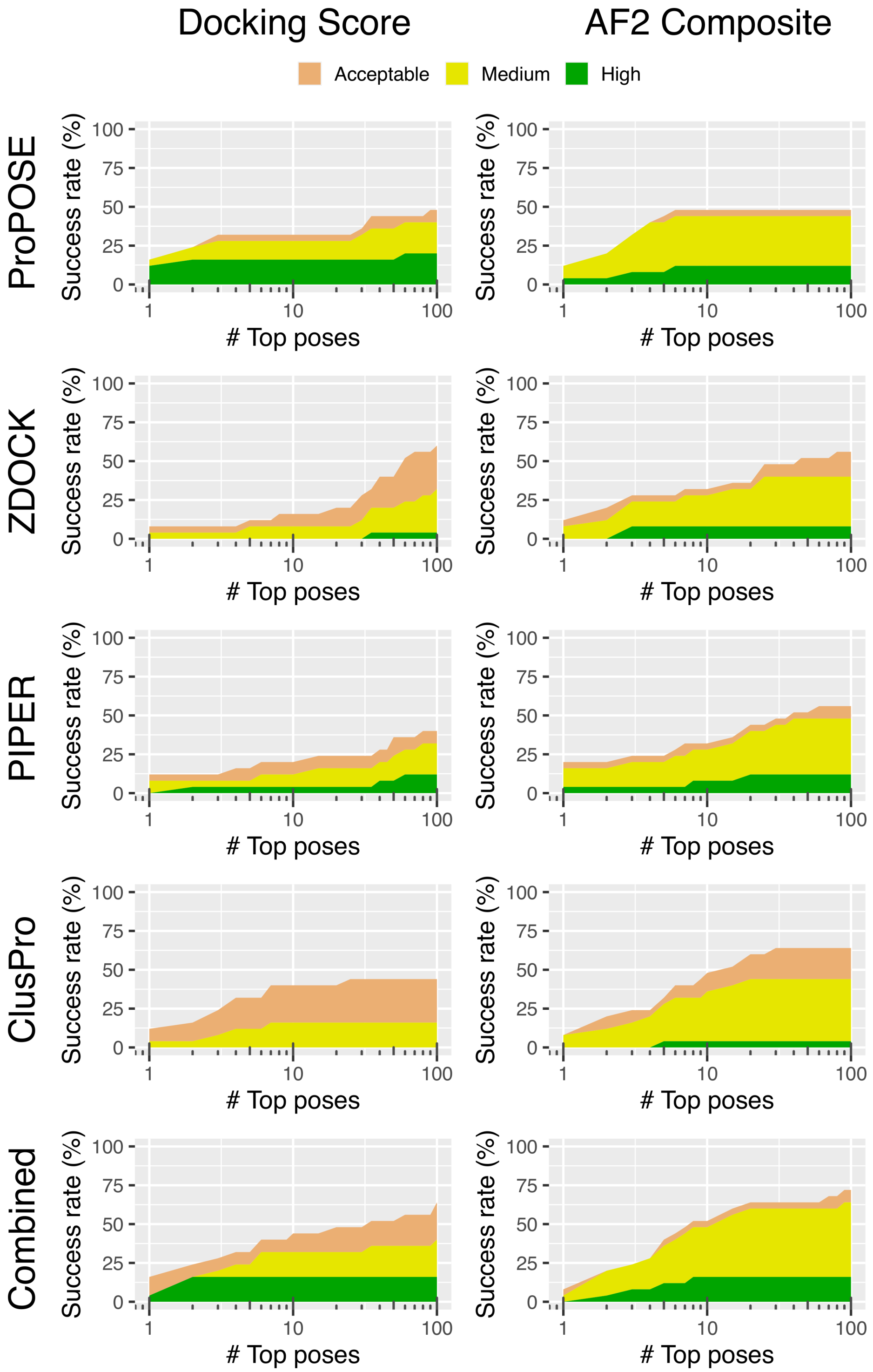

Figure S4. Success rates broken down for each model quality (see Methods) for the docking methods as a function of the number of top models considered from the ranked-list. The rates are shown for models ranked using the docking scores and the $AF2_{Composite}$ score for the unbound-backbone set. The rates were plotted on a logarithmic scale for better visibility. The success was evaluated using the docking-generated (left) and AF2-generated (right) models relative to the corresponding crystal structure.

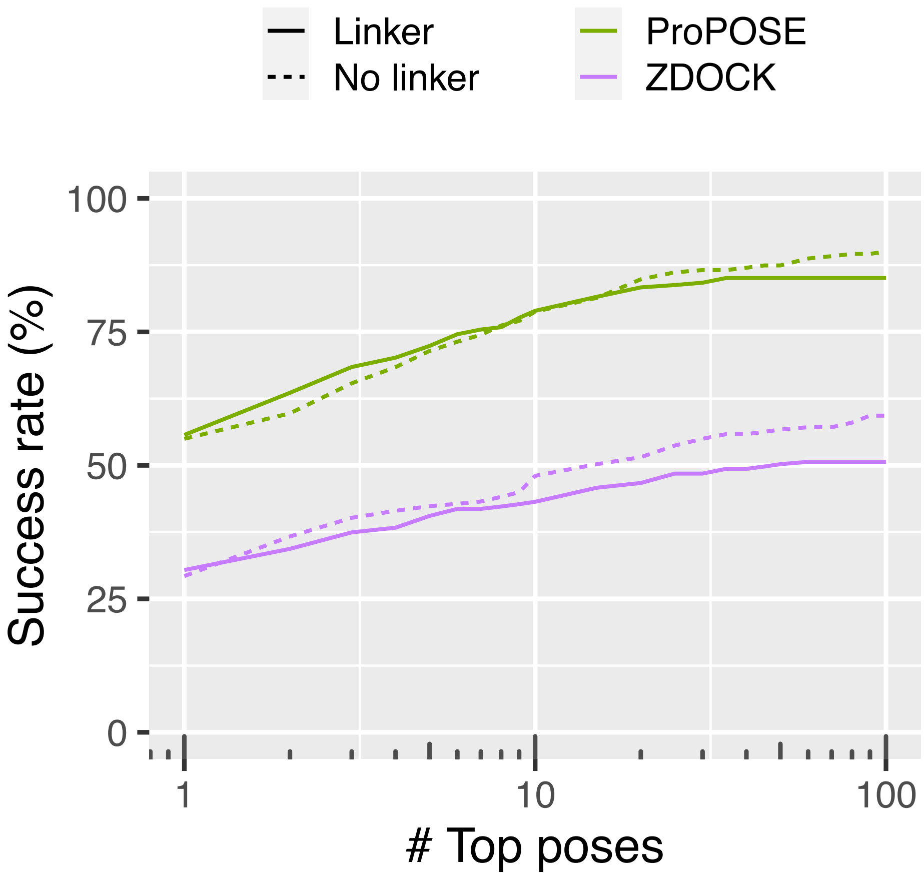

Figure S5. Influence of joining the protein chains using a 50-residue-long artificial linker (Linker) or using a 200-residue-long indexing gap (No linker) on the success rates of ProPOSE and ZDOCK on the bound-backbone set.

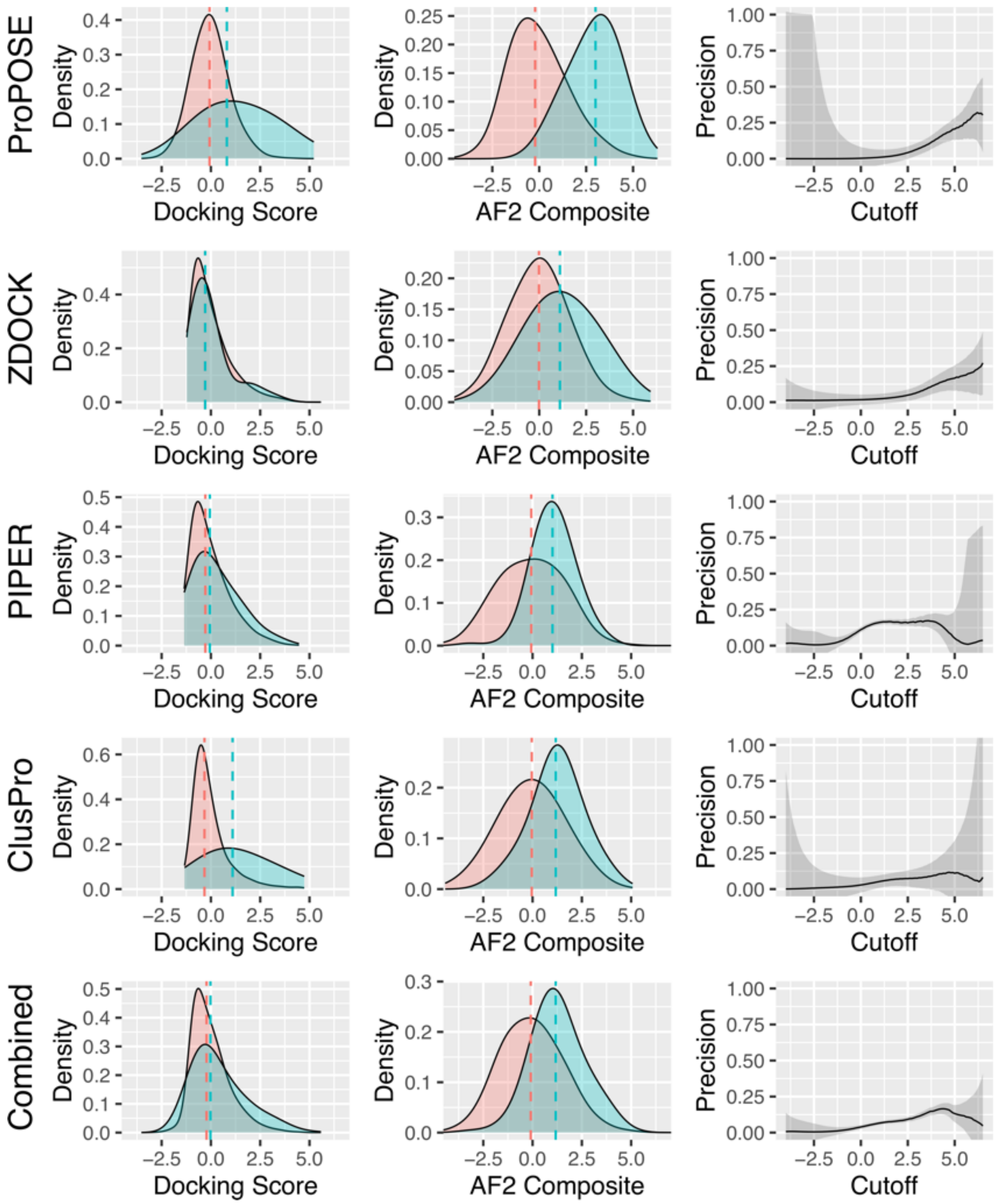

Figure S6. Density plots for the docking scores and the AF2$_{Composite}$ score for the negative and positive sets from the unbound-backbone set. The medians of the distributions are marked with dashed lines. Precision curves were built from calculating the fraction in the number of true positive over the total number of true positives and false positives according to a given cutoff in the AF2$_{Composite}$ score. The smoothed densities were used to build the precision curves to avoid outlier bias. Shadings around the precision curves indicate the errors on these curves, which were estimated based on the number of data points, i.e. less data points incur larger errors.

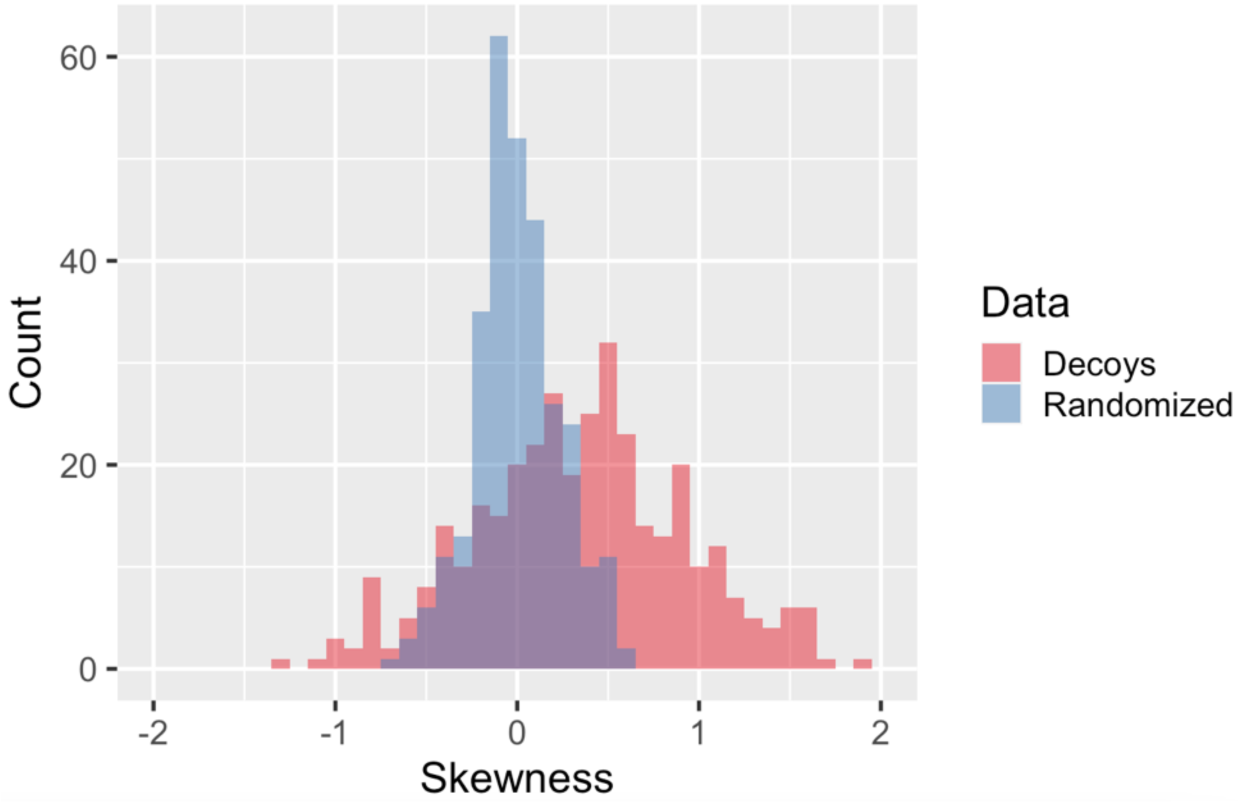

Figure S7. Skewness of distribution for the normalized AF2$_{Composite}$ scores for the individual systems in the decoys sets (Decoys) in comparison to randomized normally-distributed data (Randomized).

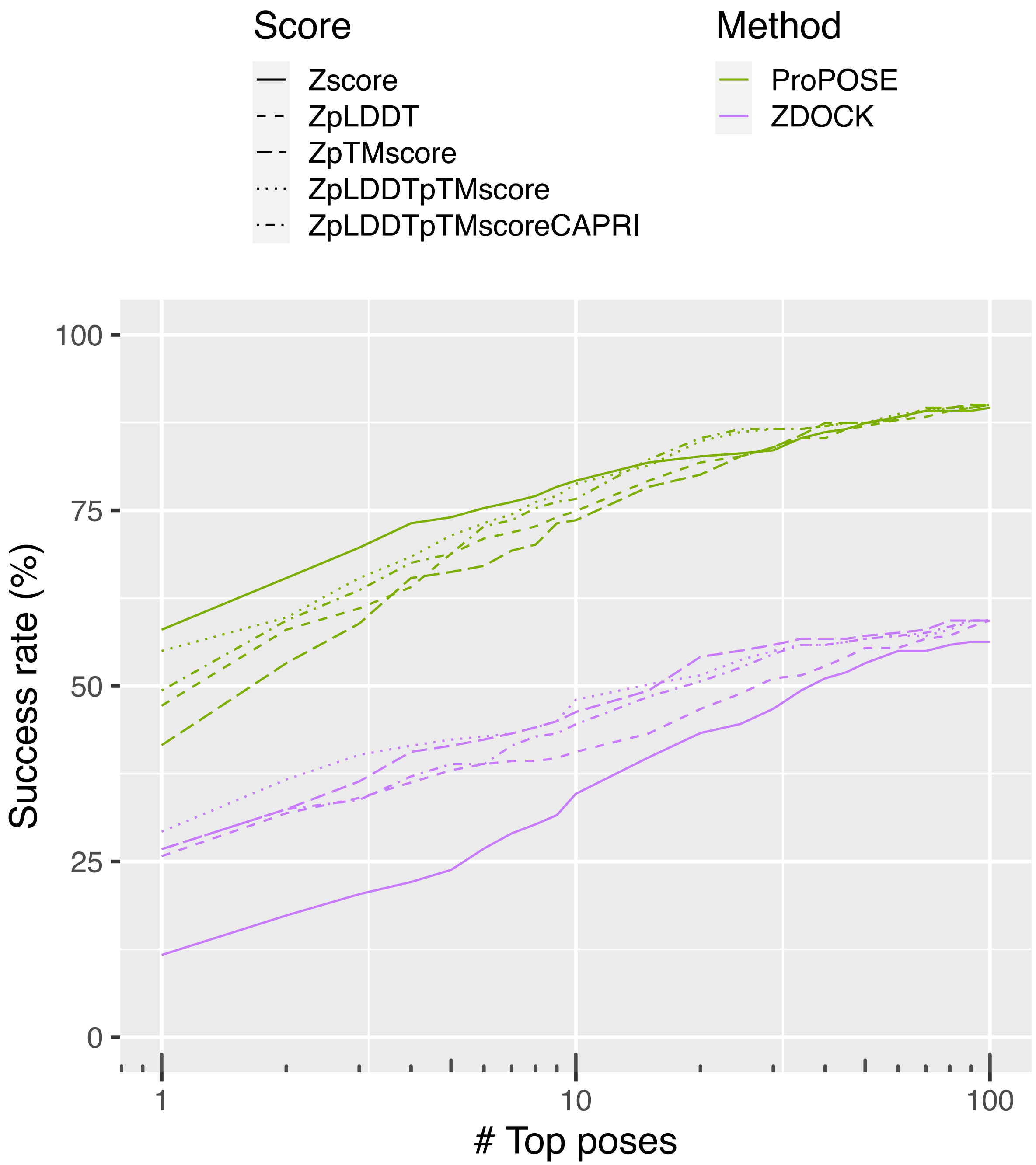

Figure S8. Influence of the scoring scheme on the success rates of ProPOSE and ZDOCK on the bound-backbone set. The scoring schemes are based exclusively on the standardized docking scores (Zscore), pLDDT (ZpLDDT), pTMscore (ZpTMscore) or from an additive combination of the pLDDT and pTMscore (ZpLDDTpTMscore) weighted by deviations of the AF2-generated model from its docking-generated template (ZpLDDTpTMscoreCAPRI). The CAPRI metrics were used as proxy to qualify the structural agreement between two structures. The weights were set at 0.25, 0.50, 0.75 and 1.00 for incorrect, acceptable, medium and high quality, respectively.

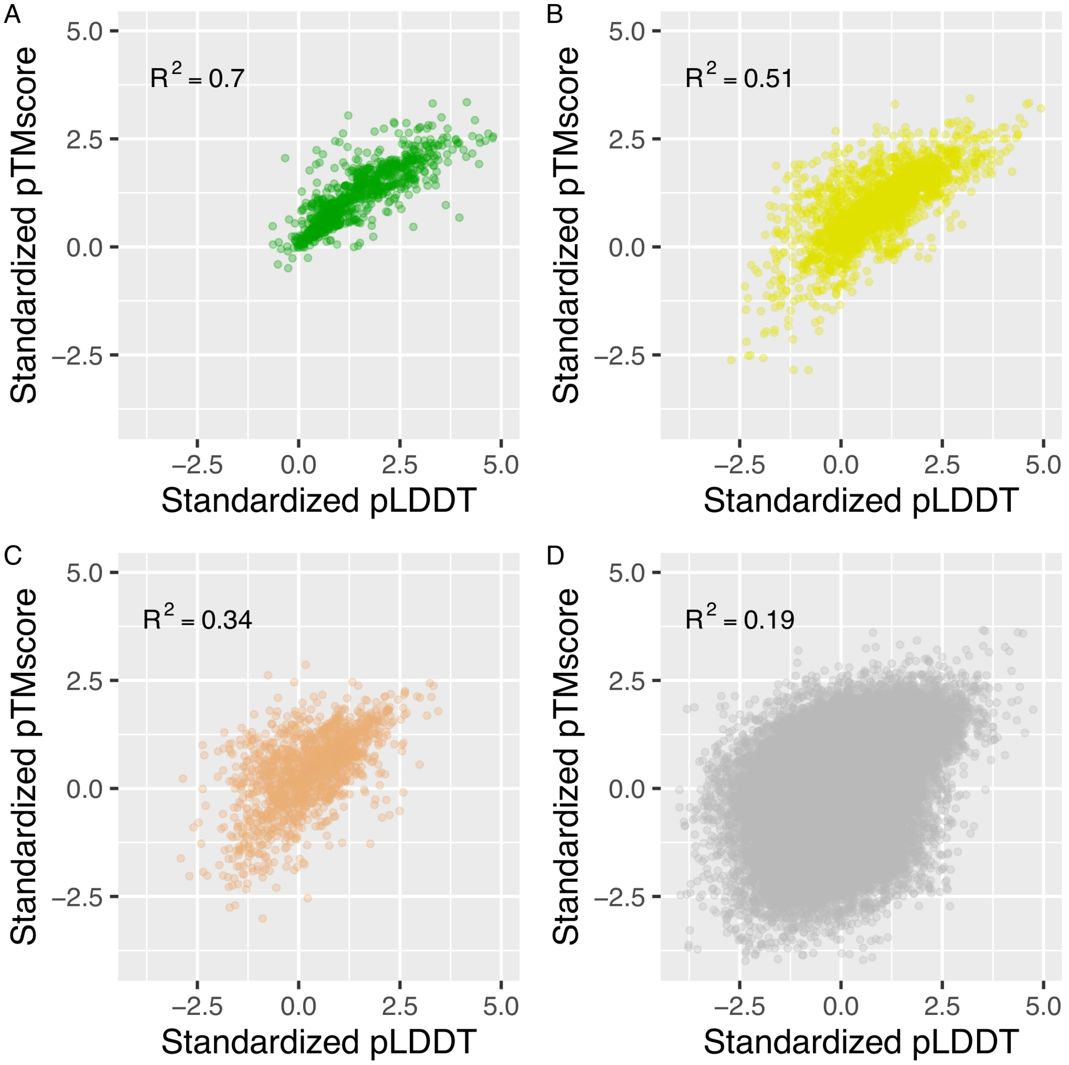

Figure S9. Correlation between the two components of the $AF2_{Composite}$ score. The scatterplots are shown separately for the four CAPRI model quality levels (see Methods): (**A**) high, (**B**) medium, (**C**) acceptable and (**D**) incorrect. All models generated by ProPOSE, ZDOCK, PIPER and ClusPro on the bound-backbone set are shown.

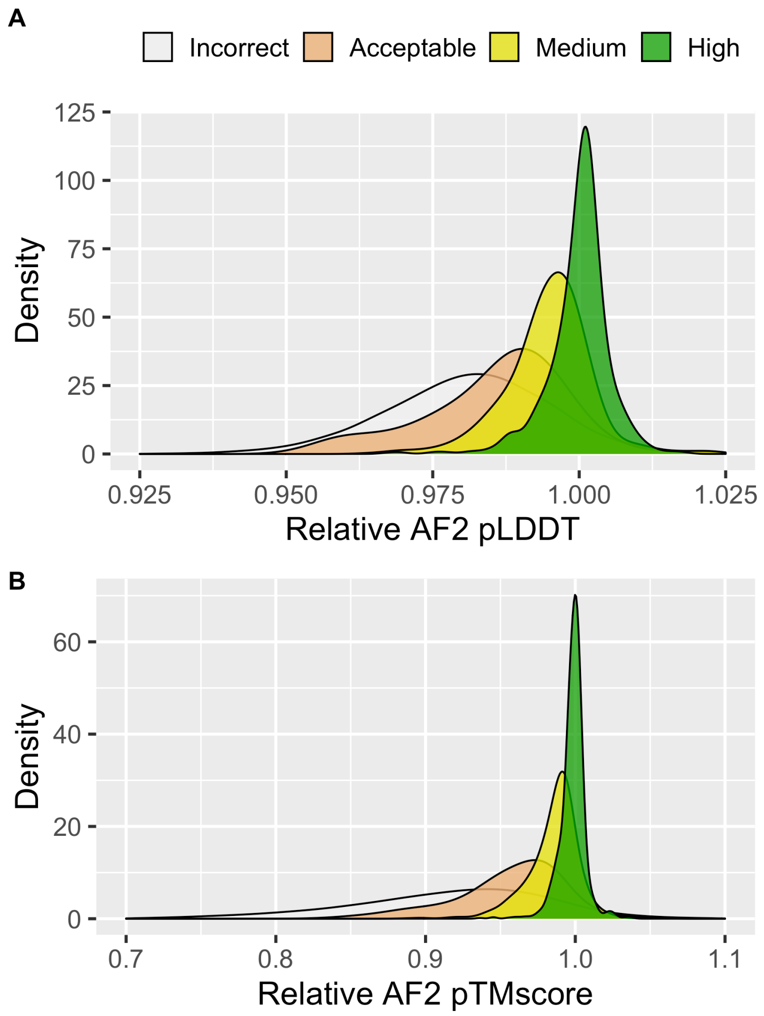

Figure S10. Smoothed density distribution of the relative AF2 confidence scores for the bound-backbone models in the negative (incorrect) and positive (acceptable-, medium- and high-quality) sets. The confidence scores are relative to those obtained by AlphaFold2 when provided with the crystal structures as input. The absolute (**A**) pTMscore and (**B**) pLDDT values are used as reference confidence scores for comparison due to the inability of deriving the composite score from a single structure in the case of the crystal. The confidence scores decrease as the quality of the model degrades.

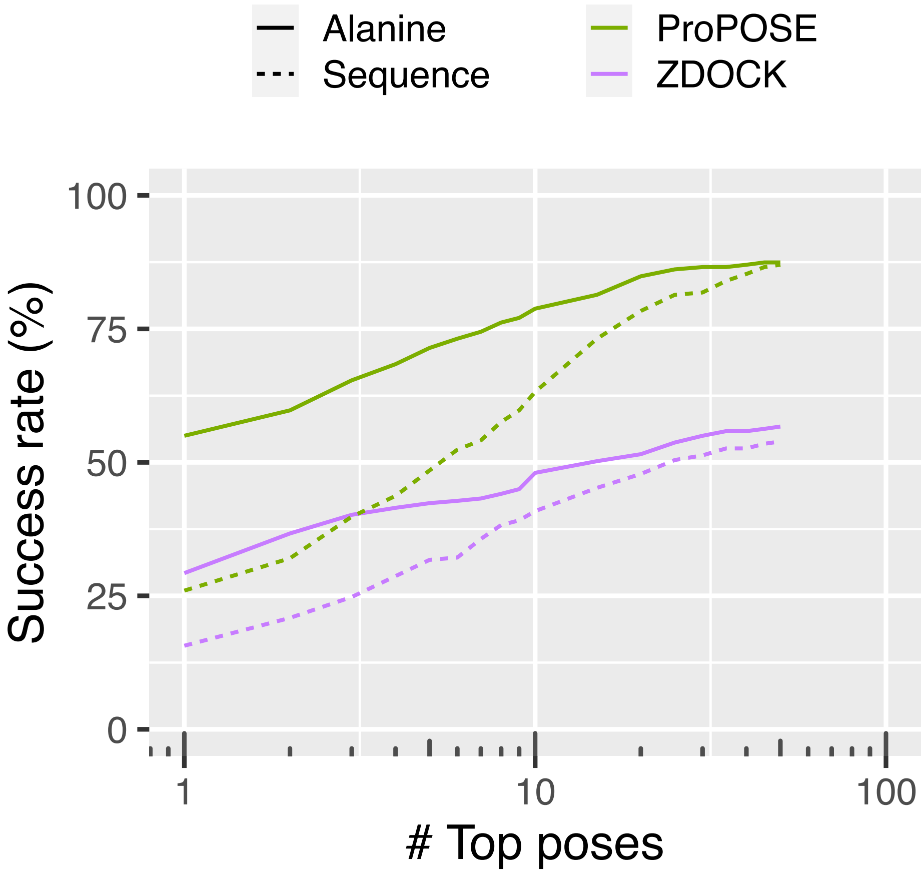

Figure S11. Influence of providing a structural template to AF2 that has its side-chains truncated (Alanine) or that has a full atomistic representation of the side-chains with preservation of the original sequence (Sequence) on the success rates of ProPOSE and ZDOCK on the bound-backbone set. Only top-50 models were rescored by AF2.

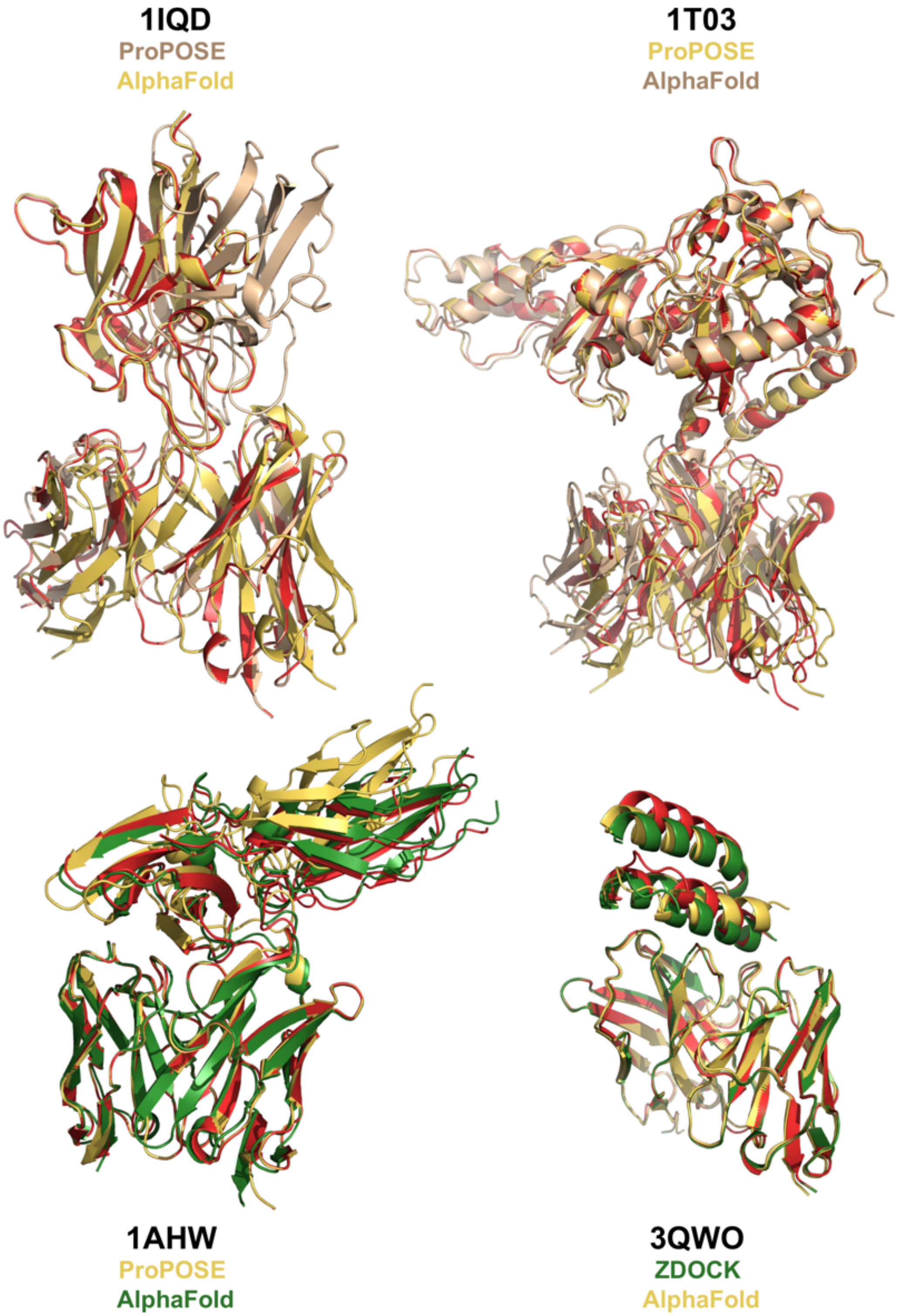

Figure S12. Illustrative examples of structural refinements by AF2 that improved (left) or deteriorated (right) the quality of docking-generated models by ProPOSE and ZDOCK. Colors denote structure quality levels as defined by CAPRI classification (see Methods): high (green), medium (yellow) and acceptable (beige). The crystal structures are shown as reference (red).

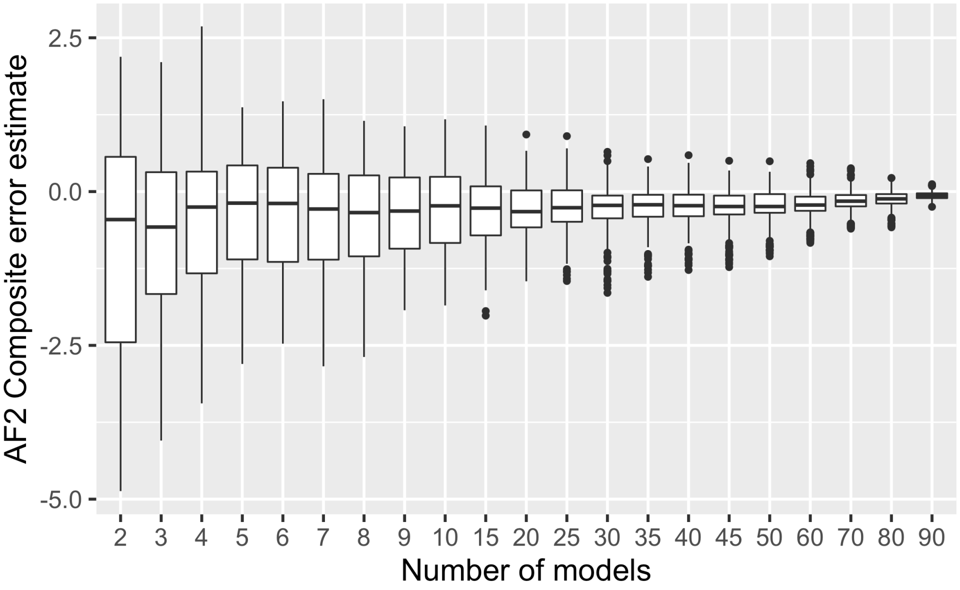

Figure S13. Error estimate on the $AF2_{Composite}$ as a function of the number of docking-generated models per system, i.e. the ensemble size. The error is calculated by subtracting the $AF2_{Composite}$ scores from models collected from smaller samples (ensembles of size below 100) to the theoretical $AF2_{Composite}$ scores from the population (ensemble of size 100). The unbound-backbone ProPOSE-generated models set were used for plotting. As the size of the ensemble increases, the estimate values approach those of the population.

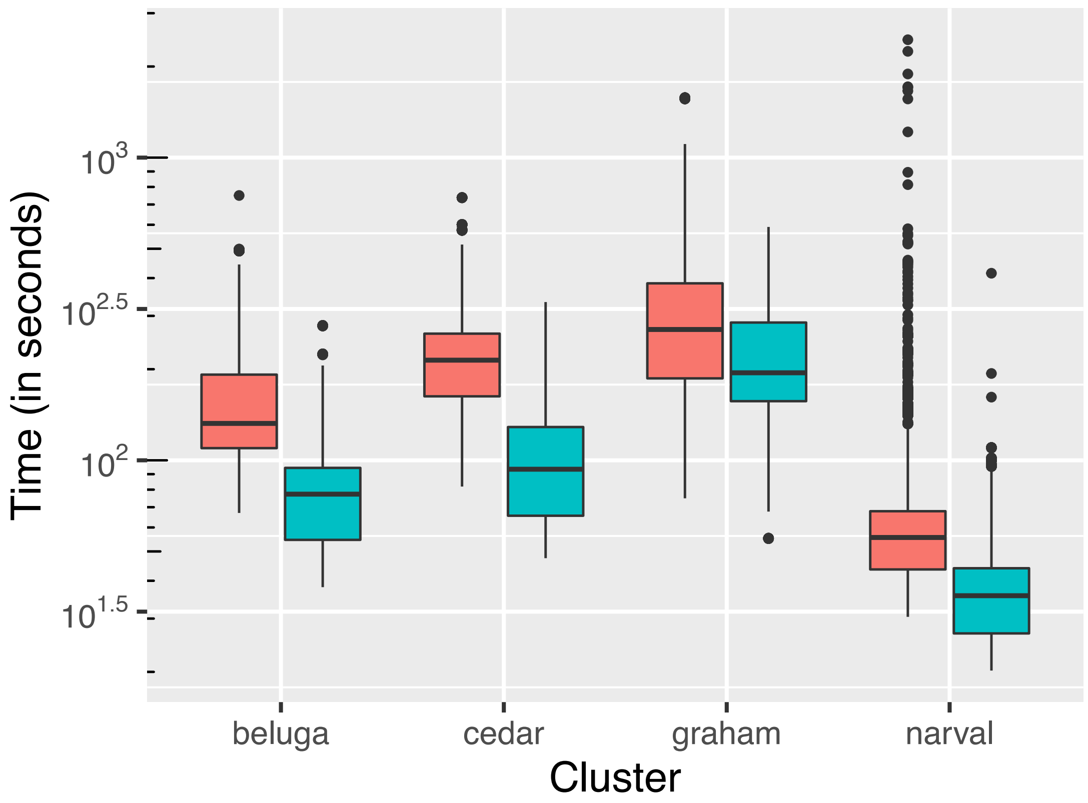

Figure S14. Runtimes per antibody-antigen model on the Compute Canada superclusters used in the computation of AlphaFold2 calculations. The clusters are exclusively comprised of A100 (narval), V100 (beluga) or are composed of a combination of V100 and P100 (cedar and graham) GPU units. The runtime is shown for AlphaFold2 using an explicit artificial-linker (red) or by introducing a residue indexing gap in the sequence (blue).

Table S1. Success rate comparison between docking-generated-template and template-free modeling of antibody-antigen complexes with medium or higher quality (CAPRI)

	Method	Top-1		Top-5		Top-10	
		Standard scoring	AF2 re-scoring	Standard scoring	AF2 re-scoring	Standard scoring	AF2 re-scoring
Bound-backbone (in AF2 training set)	ProPOSE	57%	54%	71%	71%	75%	77%
	ZDOCK	8%	27%	19%	40%	26%	42%
	PIPER	12%	31%	17%	35%	20%	37%
	ClusPro	9%	25%	18%	37%	22%	43%
	Combined	36%	44%	65%	65%	68%	74%
	AF2-Multimer	N/C [a]	N/A [b]	N/C	N/A	N/C	N/A
Unbound-backbone (in AF2 training set)	ProPOSE	16%	12%	28%	40%	28%	44%
	ZDOCK	4%	8%	8%	24%	8%	28%
	PIPER	8%	16%	8%	20%	12%	28%
	ClusPro	4%	8%	12%	28%	16%	36%
	Combined	4%	4%	24%	36%	32%	48%
	AF2-Multimer	N/C	N/A	N/C	N/A	N/C	N/A
Bound-backbone (in AF2 test set)	ProPOSE	66%	62%	75%	75%	82%	80%
	ZDOCK	11%	34%	18%	40%	25%	46%
	PIPER	18%	36%	26%	41%	31%	42%
	ClusPro	10%	31%	20%	38%	27%	40%
	Combined	47%	55%	70%	71%	79%	77%
	AF2-Multimer	11%	N/A	14%	N/A	N/M [c]	N/A

[a] N/C: not calculated due to bias in assessing AF2 performance based on structures present during its development phase; [b] N/A: not applicable; [c] N/M: not calculated due to 5-models limit of AF2-Multimer.

Table S2. Success rate comparison between docking-generated-template and template-free modeling of antibody-antigen complexes with high quality (CAPRI)

	Method	Top-1		Top-5		Top-10	
		Standard scoring	AF2 re-scoring	Standard scoring	AF2 re-scoring	Standard scoring	AF2 re-scoring
Bound-backbone (in AF2 training set)	ProPOSE	50%	31%	65%	41%	68%	44%
	ZDOCK	3%	10%	6%	14%	9%	17%
	PIPER	9%	15%	13%	21%	16%	23%
	ClusPro	1%	6%	3%	8%	4%	9%
	Combined	33%	21%	55%	39%	60%	44%
	AF2-Multimer	N/C [a]	N/A [b]	N/C	N/A	N/C	N/A
Unbound-backbone (in AF2 training set)	ProPOSE	12%	4%	16%	8%	16%	12%
	ZDOCK	0%	0%	0%	8%	0%	8%
	PIPER	0%	4%	4%	4%	4%	8%
	ClusPro	0%	0%	0%	4%	0%	4%
	Combined	4%	0%	16%	12%	16%	16%
	AF2-Multimer	N/C	N/A	N/C	N/A	N/C	N/A
Bound-backbone (in AF2 test set)	ProPOSE	56%	25%	68%	35%	75%	40%
	ZDOCK	2%	10%	3%	13%	7%	13%
	PIPER	11%	12%	18%	13%	23%	14%
	ClusPro	0%	7%	3%	8%	3%	8%
	Combined	38%	20%	59%	32%	69%	37%
	AF2-Multimer	5%	N/A	5%	N/A	N/M [c]	N/A

[a] N/C: not calculated due to bias in assessing AF2 performance based on structures present during its development phase; [b] N/A: not applicable; [c] N/M: not calculated due to 5-models limit of AF2-Multimer.
